# Paving the way or sharing goods?—Interactions between pairs of *Staphylococcus aureus* and *Pseudomonas aeruginosa* sequentially isolated from respiratory samples of patients on mechanical ventilation

**DOI:** 10.3389/fmicb.2026.1798383

**Published:** 2026-04-15

**Authors:** John Erlingsen, Dmytro Sokol, Oleksandr Ilchenko, Meissiner Gomes-Fernandes, Olena Rzhepishevska, Cristina Prat-Aymerich, Henrik Antti, Alicia Lacoma, Madeleine Ramstedt

**Affiliations:** 1 Department of Chemistry, Umeå Centre of Microbial Research, Umeå University, Umeå, Sweden; 2 Department of Microbiology, Hospital Universitari Germans Trias i Pujol, Institut d’Investigació en Ciències de la Salut Germans Trias i Pujol, Universitat Autònoma de Barcelona (UAB), Badalona, Spain; 3 CIBER Enfermedades Respiratorias, CIBER, Instituto de Salud Carlos III, Badalona, Spain; 4 Julius Center for Health Sciences and Primary Care, University Medical Center Utrecht, Utrecht University, Utrecht, Netherlands

**Keywords:** clinical isolates, co-culture, medical device, metabolomics, multi-species biofilms, *Pseudomonas aeruginosa*, *Staphylococcus aureus*

## Abstract

**Introduction:**

Bacterial colonization of medical devices is promoting hospital-acquired infections leading to worsening patient outcomes and high costs for society. Sequential bacterial colonization of surfaces may provide altered conditions that benefit pathogens.

**Methods:**

In this study we have investigated the interactions between two pairs of clinical isolates collected from patients that were on mechanical ventilation. Two patients were first colonized by *Staphylococcus aureus* and thereafter *Pseudomonas aeruginosa* settled. The two *P. aeruginosa* isolates were weak colonizers in monoculture. We investigated two hypotheses: (1) *S. aureus* preconditions material surfaces, facilitating adhesion of later colonizers. (2) *S. aureus* provides an altered nutrient environment promoting the growth and settlement of other bacteria.

**Results:**

Surface preconditioning did not seem to enhance colonization of *P. aeruginosa*. However, bacterial growth, biofilm formation, ratio of colony forming units, and metabolic profiles were influenced by co-cultivation. The effects varied depending on nutrient content in the medium.

**Discussion:**

In general, co-cultures appeared to benefit clinical isolates to a higher degree, compared to reference strains. The results indicate that differences in airway microenvironment between patients may have a large effect on the infection process and which pathogens that persist.

## 1 Introduction

Ventilator-associated pneumonia (VAP) and other ventilator-associated infections are among the most frequent acquired infections in intensive care (Papazian et al., 2020). They complicate patient care and often prolong both hospital stays and the duration of mechanical ventilation (Papazian et al., 2020). To enable early diagnosis and treatment using antibiotics, mechanically ventilated patients are therefore routinely screened for infections in the lower airways with repeated sampling of respiratory samples, such as tracheal aspirates (Papazian et al., 2020). There is a lack of consensus regarding repeated sampling, but guidelines underline the importance of clinical assessment linked to antibiotic treatment regimens (Torres et al., 2017). No clear golden standard exists for diagnosis, and treatment needs to delicately balance prevention with the risk for emergence of antibiotic resistance. Two very prevalent bacteria associated with medical devices in the airways are *Pseudomonas aeruginosa* (*Pa*) and *Staphylococcus aureus* (*Sa*) (Papazian et al., 2020; Paling et al., 2017; García-Leoni et al., 2010). The European Centre for Disease Prevention and Control stated in their surveillance report published in 2024 that 66% of ICU-associated pneumonia were linked to intubation. The top three organisms isolated were Pa (20%), Klebsiella spp. (19%) and *Sa* (18%) (European Centre for Disease Prevention and Control, 2024). These two bacteria are also responsible for a range of other types of co-infection for example in wounds and in the lung, especially in cystic fibrosis patients (DeLeon et al., 2014; Nguyen and Oglesby-Sherrouse, 2016; Yung et al., 2021). In wounds, several reports describe a spatial separation with more *Pa* deep into the wound and *Sa* close to the surface. However, the organization of the two species in lung infections have been much more challenging to investigate using histology (Camus et al., 2022), and they have been suggested to co-aggregate at early stages of infection but separate with time due to competition for resources (Alves et al., 2018). In the specific disease state of cystic fibrosis, bacteria reside in the lungs for very long periods of time, often years (VanDevanter et al., 2024; Ong and Ramsey, 2023). The patients are also undergoing repeated treatment, including antibiotics, creating a strong selection pressure. Therefore, *Pa* in cystic fibrosis lungs have been shown to undergo adaptive evolution that optimizes their fitness in the specific conditions arising in these lungs (Marvig et al., 2015). Also *Sa* strains have been shown to adapt to the cystic fibrosis lung environment (Long et al., 2021). In the *Pa* genome, mutation hotspots have been identified that give high mutation rates and contribute to that the fitness of the pathogen is optimized. Mutations have been reported in a range of functions included what types of substances the bacteria secrete, their cell wall composition, motility and antibiotic susceptibility and resistance (Marvig et al., 2015). Thus, the airways of cystic fibrosis patients are different as a growth niche compared to healthy airways, and clinical isolates from cystic fibrosis may have very different phenotypes from pathogens infecting patients without cystic fibrosis. A wealth of studies investigates pathogens and their interactions in the clinical case of cystic fibrosis (Wang Z. et al., 2025; Cendra et al., 2019; Magalhães et al., 2021; Baldan et al., 2014; Kvich et al., 2022; Limoli Dominique et al., 2017; Camus et al., 2021). However much less research has been invested in airways not suffering from this disease. In both types of airways, *Sa* and *Pa* are commonly isolated during infection symptoms in patients. The two species are among a group of bacterial species highlighted for often carrying antibiotic resistance and causing difficult infections in health care settings, the ESKAPE group (Miller and Arias, 2024). Both bacteria have previously been studied in cocultures with the aim to better understand in which ways they interact with each other and what consequences such interactions may have for patient outcome (Camus et al., 2022; Gomes-Fernandes et al., 2022). Under many conditions, both species appear to benefit from the co-localization (Camus et al., 2022; Yung et al., 2021). However, it has also been described that *Pa* often outcompetes *Sa.* A lot of work has been invested in analyzing these interactions and much has been described, however, the mechanisms are not yet fully understood for all conditions (Camus et al., 2022). It has been suggested that they take place via different routes depending on the microenvironment and conditions for growth (Yung et al., 2021; Meyer et al., 2023). The hypothesized effect of microenvironment highlights the need for controlled comparative studies where individual parameters can be varied independently, as can be done *in vitro,* in order to increase knowledge about possible interactions that may occur *in vivo*.

Competition has previously been described *in vitro* between *Sa* and *Pa* isolated from airways from VAP (Gomes-Fernandes et al., 2022). These have included growth reduction or inhibition of *Sa* by *Pa* by secreted products, such as phenazine pigments (Gomes-Fernandes et al., 2022). Coculture has been described to increase quorum sensing in *Pa* resulting in antagonism against *Sa*, but the same effects were not observed after exposure to secreted substances in culture supernatants (Gomes-Fernandes et al., 2022) indicating that some form of signaling between cells was required for the antagonism. In line with these results, 2-N-heptyl-4-hydroxyquinoline N-oxide (HQNO) has been described to target the electron transport chain of *Sa* resulting in decreasing colony sizes in presence of *Pa* cells (Yung et al., 2021). Growth of the *Pa* strains were generally not affected in mixed cultures, but growth of *Sa* strains was reduced substantially (Gomes-Fernandes et al., 2022) and lower fractions of *Sa* cells were observed in biofilms of cocultures (Camus et al., 2022). The inhibitory effect on *Sa* by *Pa* has been reported to be strain specific (Gomes-Fernandes et al., 2022) but also influenced by the microenvironment. *Pa* have been reported to outcompete *Sa* during nutrient deficiency (Nguyen and Oglesby-Sherrouse, 2016; Gomes-Fernandes et al., 2022). *Pa* has also been reported to lyse *Sa* to be able to retrieve intracellular Fe (Mashburn et al., 2005). Other reports have described that *Pa* exhibits an increased motility in presence of *Sa* facilitating invasion and formation of dual-species biofilms (Gomes-Fernandes et al., 2022; Limoli et al., 2019; Camus et al., 2022). *Sa* have been described to be a better surface colonizer which may explain why it pioneers the infection site (Camus et al., 2022). After the material surface has been colonized by *Sa*, the surface is altered by substances secreted by the bacteria, and this may facilitate for other bacteria to settle down (Kimkes and Heinemann, 2020). In this way the primary colonizer can be seen as preconditioning the surface for the late arrivals.

This work aims to investigate the hypothesis that *Sa* facilitates colonization of *Pa* under conditions of mechanical ventilation. To this aim, two pairs of anonymized clinical isolates were used originating from two patients under mechanical ventilation in an intensive care unit (ICU). These patients did not have cystic fibrosis and were first colonized by *Sa* and later by *Pa*. The *Pa* strains of these two pairs were previously investigated with respect to their colonization and biofilm forming ability on polymeric materials and were found to be weak colonizers in monoculture (Rzhepishevska et al., 2018). To investigate which factors may have facilitated sequential colonization of *Pa*, we investigated two different scenarios: (1) Sequential colonization of *Pa* could be facilitated by altered material surface properties, i.e., surface preconditioning, induced by the primary colonizer, *Sa*; (2) *Pa* could benefit metabolically by the presence of *Sa*, for example by feeding on metabolic products released from *Sa* into the microenvironment, or using *Sa* as a nutrient resource (Yung et al., 2021; Mashburn et al., 2005). This second scenario was investigated in three different nutrient conditions with and without Fe, to enable studies of different metabolic interactions between the two species in presence and absence of a key nutrient such as Fe. The study has the form of a case study, where two pairs of sequentially isolated pathogens were investigated. The study was data driven, describing how bacterial interactions may have influenced biomass accumulation at a surface as well as metabolic patterns of *Sa* and *Pa*. The study provides a stepping stone for future more detailed studies of specific metabolic mechanisms linked to bacterial cohabitation.

## 2 Materials and methods

### 2.1 Clinical isolates and reference bacteria

The bacterial strains were isolated from tracheal aspirate samples from two patients under mechanical ventilation admitted at the ICU of Hospital Germans Trias, Badalona, Spain. Ethical approval was provided and the need for informed consent was waived by the Institutional Review Board: Comite d’Etica de la Investigacio de l’Hospital Germans Trias i Pujol. Patient consent was waived because material used were strains and clinical data were anonymized and the isolated strains did not contain host material that can be traced to any person. Sample collection followed the standard of care and was based on suspicion of infection, and therefore, was not performed on the same days for the two patients. Patients clinical data, and antimicrobial susceptibility profile for the 4 isolates are found in Supplementary Tables 1, 2. During microbiological investigations, well-isolated individual colonies were picked up, subcultured and thereafter stored at −80 °C. The first pair of isolates from one individual patient is here labeled A. This pair included an initial colonizer, *S. aureus* called SaA. In a subsequent bacterial culture after 11 days, this strain had been replaced by a strain of *P. aeruginosa* which remained present also at 24 days after the isolation of SaA. This second isolate was collected 24 days after the initial isolation of SaA and was labeled PaA (CCGU71613). It is not known if these two bacterial isolates (SaA and PaA) had, at any time, been simultaneously present in the patient. The second pair, labeled B, consisted of an initial colonizer: *S. aureus*, SaB, that remained in the patient at a second sampling after 7 days and was, at that time, co-isolated with *P. aeruginosa,* PaB, (CCUG71609). After 10 additional days, SaB was no longer observed in this second patient, and the culture only contained PaB. The two *P. aeruginosa* isolates (PaA and PaB) were previously studied as monocultures (Rzhepishevska et al., 2018) but not in combination with other bacteria. In addition to the clinical strains, two reference strains were included in this study: *S. aureus* Newman (Baba et al., 2008) (SaN) and *P. aeruginosa* PA01 (Pa01) to facilitate future comparisons with other studies. Colony morphology of the isolates was recorded on blood-agar plates and is shown in Supplementary Figure 1. In this work, from now on, *Pa* or *Sa* will be used to refer to the species while SaN, SaA, SaB, Pa01, PaA and PaB will refer to the specific strains used.

### 2.2 Culture media

All chemicals were purchased from Merck and used without further purification, unless specifically stated otherwise. Bacterial culture media were based on diluted tryptic soy broth (TSB) supplemented with glucose (Gomes-Fernandes et al., 2022). Three types of media were prepared based on TSB diluted with Milli-Q water (MQ) to 50% (v/v) (0.5xTSB). Medium 1 (M1) was TSB supplemented with 1% (w/v) glucose. Medium 2 (M2) was Fe-free TSB also supplemented with glucose. Medium 3 (M3) was Fe-free TSB. Fe-free media were prepared from 0.5xTSB using Chelex 100 resin (Bio-Rad) to remove metal ions. Thereafter, essential salts (excluding Fe) were added as follows: 0.55 mM MgSO_4_, 1.5 μM ZnSO_4_, 0.2 μM CuSO_4_, 1 μM MnCl_2_, 5 μM CaCl_2_ (Lindgren et al., 2015). The salts were dissolved in MQ water and filtered through a 0.2 μm filter before addition to the Chelex-treated base medium. M2 represents as an intermediate nutrient composition between M1 and M3.

### 2.3 Bacterial initial attachment assays

Initial adhesion of bacterial cells was investigated using a previously published method (Rzhepishevska et al., 2018). In short, mid-log-phase cells were suspended in NaCl (0.9%) to optical density at 600 nm (OD_600_) of 1.0. Thereafter cell suspensions were pipetted into wells of 96-well plates and incubated at 37 °C for 30 min. The wells were carefully emptied, and the attached cells were stained using crystal violet. The dye was eluted using 33% acetic acid and absorbance of crystal violet (CV) bound to attached cells was analyzed at 595 nm in a plate reader (Wallac Victor, Perkin Elmer, United States). Three preconditioning cases were tested: (1) 30 min initial attachment of *Sa,* careful rinsing with 0.9% NaCl, followed by 30 min *Pa* attachment; (2) preconditioning of culture wells for 24 h at 4 °C with filtered supernatant from *Sa* culture before attachment of *Pa*. The preparation of the cell-free supernatants was carried out as previously described (Gomes-Fernandes et al., 2022). (3) Preconditioning of wells using culture of *Sa* allowed to grow and form biofilm in the wells for 24 h at 37 °C, thereafter the wells were carefully rinsed with 0.9% NaCl before 30 min attachment of *Pa*.

### 2.4 Growth curves and viable counts

Bacteria were cultivated at 37 °C overnight on Miller Hilton (MH) agar plates. For each strain, colonies were taken with a sterile loop, resuspended in sterile culture medium broth and adjusted to OD_600_ = 0.5. Cocultures were prepared by mixing bacterial suspensions in a 1:1 ratio to a total OD_600_ = 0.5. Thereafter, the suspensions were diluted to OD_600_ = 0.1, and from this suspension 20 μL were added to 180 μL pure media in flat-bottom 96-well plates (standard non-coated microplates, Sarstedt). Wells with only medium served as sterility controls. Wells at the edges and corners were filled with water to prevent errors related to evaporation. Each data point represented an average of at least 6 wells. Growth curves for the three types of media were recorded from the 96-well plates incubated statically at 37 °C, and OD_600_ measurements were done using a plate reader (Perkin Elmer Wallac 1420 Victor) at room temperature at 0 h, 3 h and 6 h, thereafter measurements were conducted every 2 h until the end of the experiment. The plates were returned to 37 °C directly after each measurement. Time intervals between plates were staggered to mitigate the impact of temperature fluctuations in the plates. To avoid condensation on the lid of the 96-well plates, Triton solution was applied to the lids during preparation.

Colony forming units (CFU) were used to quantify the number of viable bacteria after 15 h cultivation at 37 °C in the three media, both in monocultures and cocultures, following the procedure described above. A total volume 100 μL of bacterial suspension was collected from two wells of a 96-well plate (50 μL + 50 μL). This sample was subsequently diluted to between 10^−5^ and 10^−6^. From these dilutions, 100 μL was plated onto blood-agar plates, spread evenly using glass beads (Sanders, 2012), and incubated overnight at 37 °C for enumeration the following day. Differential morphology on blood-agar plates enabled the enumeration of both species from the same plates (Supplementary Figure 1).

### 2.5 Static biofilm formation after 20 h

To study biofilm formation, monocultures of clinical and control *Sa* (SaA, SaB and SaN), *Pa* (PaA, PaB and Pa01) as well as cocultures (SaA & PaA, SaB & PaB, Pa01 & SaN) were used. Overnight cultures were prepared from MH agar plates. Bacterial biomass was resuspended in 0.9% (w/v) NaCl (high-purity grade, VWR Chemicals, United States) to a final OD_600_ = 1.0 and diluted with NaCl solution to a final OD_600_ = 0.1. Wells of standard non-coated 96-well plates were filled with 180 μL medium. Corner and edge wells were filled with 200 μL of sterile 0.9% (w/v) NaCl to reduce errors due to evaporation during incubation. Thereafter, 20 μL of bacterial suspension was transferred to 96-well plates, resulting in OD_600_ = 0.01 as a starting point (6 replicates per sample). For cocultures, the *Sa* and *Pa* ratio was 1:1 by volume. The 96-well plates were incubated statically at 37 °C for 4 h. To provide fresh nutrient medium, 150 μL of respective liquid media from each well was discarded carefully after 4 h, without disturbing the formed biofilm and 150 μL of fresh respective warm media (37 °C) was carefully added into each well. After that, all 96-well plates were incubated for an additional 16 h at 37 °C. The crystal violet (CV) staining biofilm assay by O’Toole and Kolter (1998) was used to quantify biofilm volume. The whole staining procedure took place at room temperature (~21 °C). All liquid in each well was carefully discarded using a pipette. To preserve the biofilm, 200 μL of 99% (v/v) ethanol was added into each well and the plate incubated for 30 min. After that, ethanol was aspirated, and wells were air dried. After 1 h drying, each well was incubated with 200 μL of a 0.1% (w/v) aqueous solution of CV for 15 min. Then, the CV solution was removed, and each well was washed twice with 200 μL of 0.9% (w/v) NaCl. The plates were air-dried overnight. The following day, 200 μL of 33% (v/v) acetic acid was used to extract the dye in each well. After 15 min incubation liquid was transferred to new 96-well plates and the quantity of dye determined in a microplate reader (CRALIOstar^®^, BMW Labtech) at a wavelength of 595 nm.

### 2.6 Metabolite analysis using gas chromatography-mass spectrometry

Samples for metabolite analyses were prepared from both monocultures and cocultures. Cocultures consisted of SaA + PaA and SaB + PaB. All samples were prepared in the three media described in section 2.2. Cultivation of the bacteria took place in the same way as for the biofilm formation experiments in section 2.5. Metabolites were separately extracted from supernatant and cell fraction of cultures grown for 15 h in 96-well plates in the three different media (M1, M2, M3). For each experimental condition (media and strain), supernatant samples for metabolite extraction were prepared in triplicates and cell samples were prepared in duplicates due to technical reasons of the sample preparation logistics. Each replicate for metabolite extraction was prepared from two independent cultures grown in 96-well plates (a pool from two cultures). For each cell sample, bacterial suspension was adjusted to OD_600_ = 0.2 with ice cold 0.9% NaCl on ice and 1.2 mL of this suspension was centrifuged at 13,000 rpm, 1 min. Cell pellet was washed once with 0.5 mL of ice-cold 0.9% NaCl metabolites from culture supernatants (50 μL) and from bacteria cell pellets were extracted with 450 μL of extraction mixture [MeOH/H_2_O (90:10 v/v)] supplemented with internal standards in separate Eppendorf tubes. A tungsten bead was added to each Eppendorf, samples were shaken in a bead mill for 2 min and placed on ice. After 2 h ice-bath incubation, samples were centrifuged for 10 min, at 4 °C, at 14,000 rpm in a tabletop centrifuge (Hettich 220-220R, Germany). Supernatant after centrifugation, (50 μL) was evaporated to dryness in GC-vials using miVac Quatro Concentrator (Gene Vac). For *Sa* cell samples cultured in three different media types and designated “c100” in the data set we additionally prepared samples where 100 μL of extracts were used as a technical control for quality of metabolite extraction from these types of cells. Methoxamine (15 μL) was added to each vial; the vial was vortexed (10 min) and incubated at 70 °C for 1 h and then at RT for 16 h. For derivatization, a 1:1 mix (30 μL) of of N-Methyltrimethylsilyl-trifluoroacetamide (MSTFA) 1% and heptane (including methyl stearate 15 ng/mL), was added, the samples shaken briefly and incubated at room temperature for 1 h before the analysis. The samples were analyzed using Pegasus^®^ BT GC-TOFMS, Benchtop Gas Chromatography Time-of-Flight Mass Spectrometer equipped with silica/DB5-MS capillary column (10 m × 0.18 mm × 0.18 μm i.d.) and helium as a mobile phase. For cell samples, sample injection volume was 1 μL and for supernatant samples the injection volume was 0.1 μL.

### 2.7 Metabolite data processing

GC-MS chromatographic raw data were preprocessed with the MATLAB-based tool provided by the Swedish Metabolomics Center (Umeå Sweden). The tool implements the methodology described in Jonsson et al. (2004) and Jonsson et al. (2005). The workflow included automatic deconvolution of co-eluted peaks to resolve overlapping signals into pure spectral profiles. Prior to deconvolution, the raw GC-MS data underwent noise reduction, baseline correction, and retention time alignment. The resolved data were searched against an electron ionization mass spectral (EI-MS) database containing 738 unique MS compounds provide by the Swedish Metabolomics Center. In addition, metabolite annotations were verified with NIST MS Search 08 software using the NIST Library, NIST/EPA/NIH Mass Spectral Library (NIST 08) which in total contains 92,559 spectra in 17 libraries. The verification process was performed using the nistAutoSearch tool^1^ (Ilchenko, 2025). Manual curation was used by individually checking metabolite resolved peaks profiles and removing those with irregular peak shapes, keeping only peaks that displayed a Gaussian-like distribution. Metabolite features detected in fewer than 50% of QC samples were excluded from further analysis. The data treatment workflow consisted of four steps: (1) missing-value imputation using the half-minimum method, (2) log₁₀ transformation, (3) mean-centering, and (4) Pareto scaling for normalization, which were used to remove systematic variation in the data not related to variation of biological interest. Output data were evaluated using both univariate and multivariate statistics. The biological interpretation of the dataset was based on models from multivariate analysis including unsupervised principal component analysis (PCA) and supervised orthogonal partial least squares discriminant analysis (OPLS-DA) performed using SIMCA 17 (Sartorius, Umeå, Sweden). Statistical significance of *p* < 0.01 for metabolite loadings (Tables 1, 2) was determined using the equal variance unpaired Students *t*-test with Benjamini–Hochberg false discovery rate correction.

**Table 1:** Supernatant metabolites driving separation in OPLS-DA models (weighted loadings driving the separation are listed, *p* < 0.01) comparing M3 and M1 medium for three types of cultures.

Metabolite	*Sa*	*Pa*	*Sa* + *Pa*
2-Aminobutyric acid	0.09		0.14
2-Deoxytetronic acid	0.15	0.12	
2-Hydroxyhexanoic acid	0.09		
3-Phenyllactic acid	0.10		
4-Aminobutyric acid	0.12		
Alanine			0.13
α-Hydroxybutyric acid	0.09		0.11
Arginine			0.14
Benzoic acid der	0.09		
Butanedioic acid der	0.14		0.13
Cholesterol	0.15	0.16	0.15
Citric acid	0.14		0.15
Erythritol	0.10		
Fructose		0.14	0.14
Glyceric acid	0.14	0.14	
Glycerol 3-phosphoate	0.11	0.13	0.13
Glycine	0.10	0.13	0.14
Hexanal			0.11
Inositol	0.13		0.14
Isoleucine	0.15	0.15	0.15
Lactose			0.08
Leucine	0.15	0.16	0.15
Lysine			0.11
Malic acid	0.13		0.09
Methionine	0.13	0.14	
Octadecanoic acid	0.15	0.15	0.14
Ornithine	0.12		0.14
Phenylalanine	0.11	0.10	0.15
Phosphoric acid	0.15	0.15	0.15
Pinitol	0.14	0.15	0.15
Proline			0.09
Putrescine	0.13	0.15	0.14
Pyroglutaic acid	0.12		0.09
Raffinose	0.15	0.15	0.14
Ribose		0.12	
Serine		0.14	0.10
Sorbose			0.15
Succinic acid	0.15	0.16	
Threonic acid	0.12		
Threonine		0.12	0.12
Tryptophan	0.13		0.14
Tyrosine	0.12		0.12
Valine	0.15	0.12	0.15
Xylose		0.13	
Galactosamine	−0.11	−0.14	−0.14
Glucose	−0.15	−0.17	−0.15
Gulose	−0.15	−0.17	−0.15
Hexose	−0.15	−0.14	−0.15
Isomaltose	−0.09	−0.15	−0.14
Lactulose	−0.11		
Ribitol		−0.12	−0.14
Ribose	−0.13		
Sucrose	−0.13		
Sucrose			−0.13
Threhalose	−0.13		−0.12
Threonic acid		−0.15	
Trehalose	−0.14		−0.13
Xylitol	−0.12	−0.15	−0.14
Xylose	−0.13		
Xylulose	−0.14		

Positive loading linked to correlation to M3 and negative loadings to M1. (Unidentified metabolites shown in Supplementary Table 3). Metabolites are given in alphabetic order. Model goodness of fit (*R*^2^*Y*) and predictive ability (*Q*^2^) were 0.988 and 0.985 for *Sa*, 0.99 and 0.984 for *Pa* and 0.991 and 0.987 for the coculture. *p*-value from CV-ANOVA 8.6 × 10^−20^ for *Sa*, 1.2 × 10^−16^ for *Pa* and 2.3 × 10^−20^ for the coculture.

**Table 2:** Bacterial cell pellet metabolites driving separation in OPLS-DA models (weighted loadings shown, *p* < 0.01) comparing M3 and M1 medium for three types of cultures.

Metabolite	*Sa*	*Pa*	*Sa* + *Pa*
2-Methyl-2-butanol		0.11	0.11
4-Aminobutyric acid	0.11		
6-Hydroxynicotinic acid	0.16		
Adenosyl-methionine			0.11
Arginine	0.13		
B-Cyano alanine	0.11	0.13	0.14
Beta-alanine	0.15		
Cystathionine			0.12
Decanoic acid		0.10	
Ethanedioic acid der		0.11	0.13
Fumaric acid	0.12		
Galactose	0.12		
Glycerol	0.15	0.14	0.15
Homoserine	0.13	0.13	0.15
Lysine	0.13		
Malic acid		0.15	0.16
Myristic acid	0.15	0.14	0.16
Nonanoic acid			0.11
Octadecanoic acid		0.12	
Oxalic acid		0.12	0.13
Phenylalanine	0.14		0.11
Ribose			0.14
Succinic acid		0.10	0.14
Sucrose			0.12
Xylibiose	0.16	0.10	0.13
4-Aminobutyric acid		−0.11	
Cadaverine		−0.11	
Fructose		−0.15	−0.17
Gluconic acid		−0.14	−0.13
Gluconic acid der	−0.15		−0.13
Glucose	−0.16		
Glutamic acid		−0.13	−0.13
Glycerolphosphate	−0.13	−0.11	
L-Aspartic acid		−0.12	
Lysine		−0.11	
Pyroglutamic acid		−0.15	−0.14
Ribitol	−0.17		
Trehalose	−0.11		
Uridine der	−0.17	−0.10	−0.13

Positive loading linked to correlation to M3 and negative loadings to M1. (Unidentified metabolites shown in Supplementary Table 4). Model goodness of fit (*R*^2^*Y*) and predictive ability (*Q*^2^) were 0.985 and 0.95 for *Sa*, 0.984 and 0.939 for *Pa* and 0.936 and 0.91 for the coculture. *p*-value from CV-ANOVA 4.7 × 10^−12^ for *Sa*, 2.0 × 10^−9^ for *Pa* and 1.1 × 10^−11^ for the coculture.

## 3 Results

Sequential colonization of *Pa* into surfaces already colonized by *Sa* may occur due to several factors. Here two scenarios were investigated using pairs of clinical strains sequentially isolated from the same patient. These two scenarios could influence colonization individually or in combination. The first scenario investigated, related to possible surface alteration of a polymeric material by *Sa* that could facilitating later colonization of *Pa* (section 3.1). The second scenario related to competitive and synergistic traits linked to bacterial metabolism in three different conditions, including Fe limitation (sections 3.2–3.3).

### 3.1 Does preconditioning increase initial bacterial attachment?

**Figure 1:**
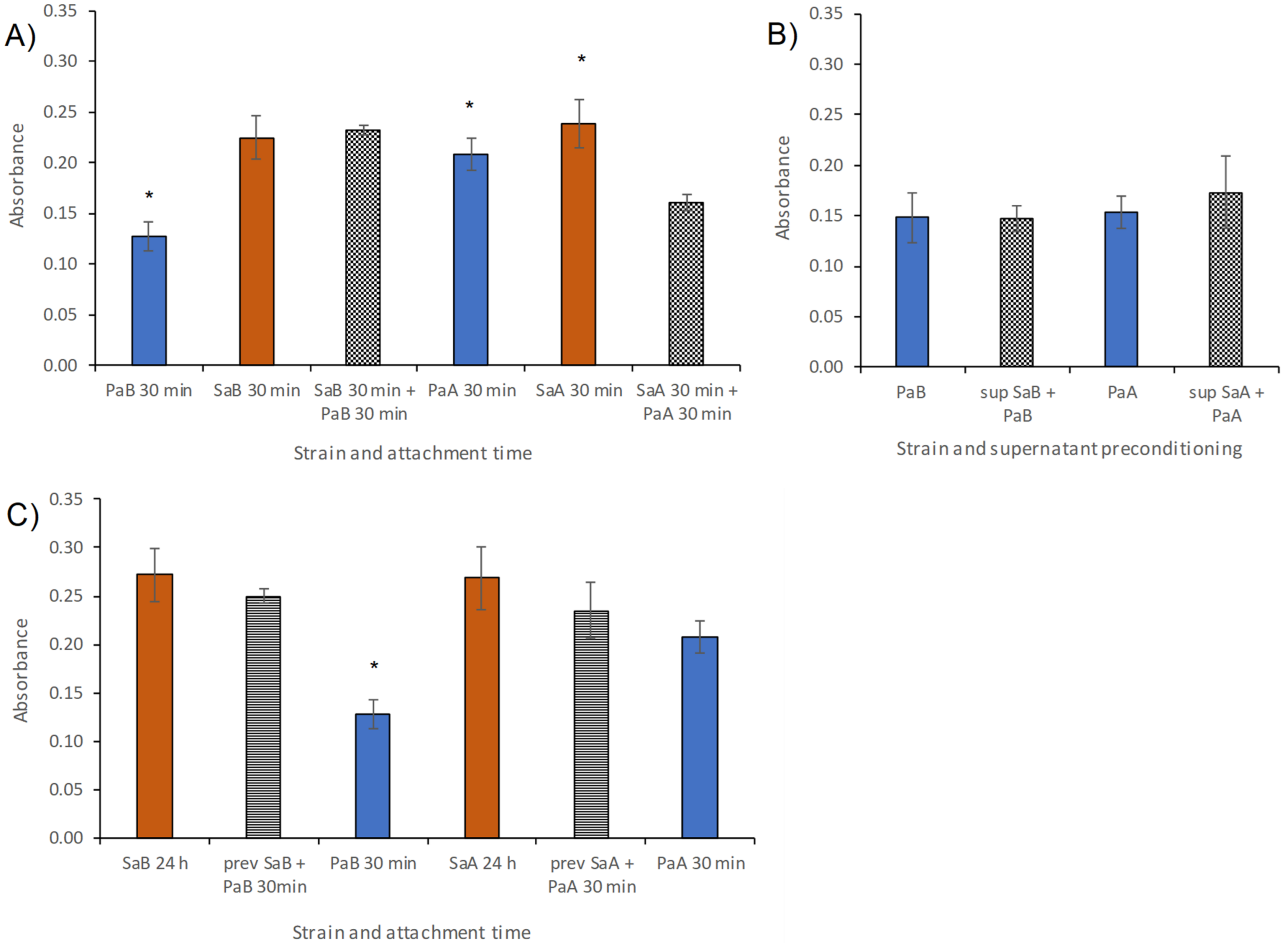
Initial attachment assays (30 min) investigating the possibility for surface preconditioning of *S. aureus* (*Sa*) facilitating attachment of *P. aeruginosa* (*Pa*). The attached biomass was determined using crystal violet staining (absorbance 595 nm). Three different forms of preconditioning were investigated: cells, supernatant and biofilm: Attachment of **(A)** PaB, SaB, PaA, and SaA (solid bars are controls, red for *Sa* and blue for *Pa*) or sequential attachment of the two (checked bars) after 30 min, all on untreated culture plastic, **(B)** attachment of PaB or PaA on pure culture plastic (solid blue bars, controls), or on culture plastic preconditioned by SaB or SaA supernatant (checked bars), **(C)** solid red bars show biofilm formation from *Sa* in 24 h culture (control), striped bars show results after 30 min sequential attachment of *Pa* following after 24 h biofilm growth of *Sa*. Solid blue bars show *Pa* attachment after 30 min (control). All bars represent average of four samples and error bars represent standard deviation. Bars with a significantly difference between preconditioning and control samples (solid bar vs. checked/striped bar), are marked with * (*p* < 0.01, students *t*-test) on the solid bar.

Preconditioning of surfaces is well known to increase initial bacterial attachment (Tuson and Weibel, 2013; Ramstedt et al., 2019). To investigate this possibility, three sets of attachment experiments were performed. First, initial attachment of the four isolates was investigated from saline (NaCl 0.9%) onto culture plastic in 24-well plates. None of the strains exhibited pronounced attachment after 30 min (OD < 0.5 Rzhepishevska et al., 2018). Some small differences in attachment were observed between the four strains where PaB attached the least giving very low reading for total biomass. Sequential attachment did not increase the biomass of attached cells (Figure 1A). Secondly, experiments were conducted in order to study possible preconditioning by substances produced and secreted by *Sa* facilitating subsequent attachment of *Pa*. For this aim, culture wells were pre-treated for 24 h with supernatant from *Sa* cultures. However, no increase in overall biomass following attachment of *Pa* cells was observed (Figure 1B). As a third step, sequential colonization was investigated by first culturing *Sa* for 24 h at 37 °C and thereafter studying increase in biomass after introduction of *Pa*. This preconditioning also did not appear to increase overall attached biomass (Figure 1C). Thus, we concluded that surface preconditioning, by itself, most likely was not the main factor giving rise to the sequential colonization.

### 3.2 How does nutrient composition affect bacterial interactions, growth and biofilm formation?

**Figure 2:**
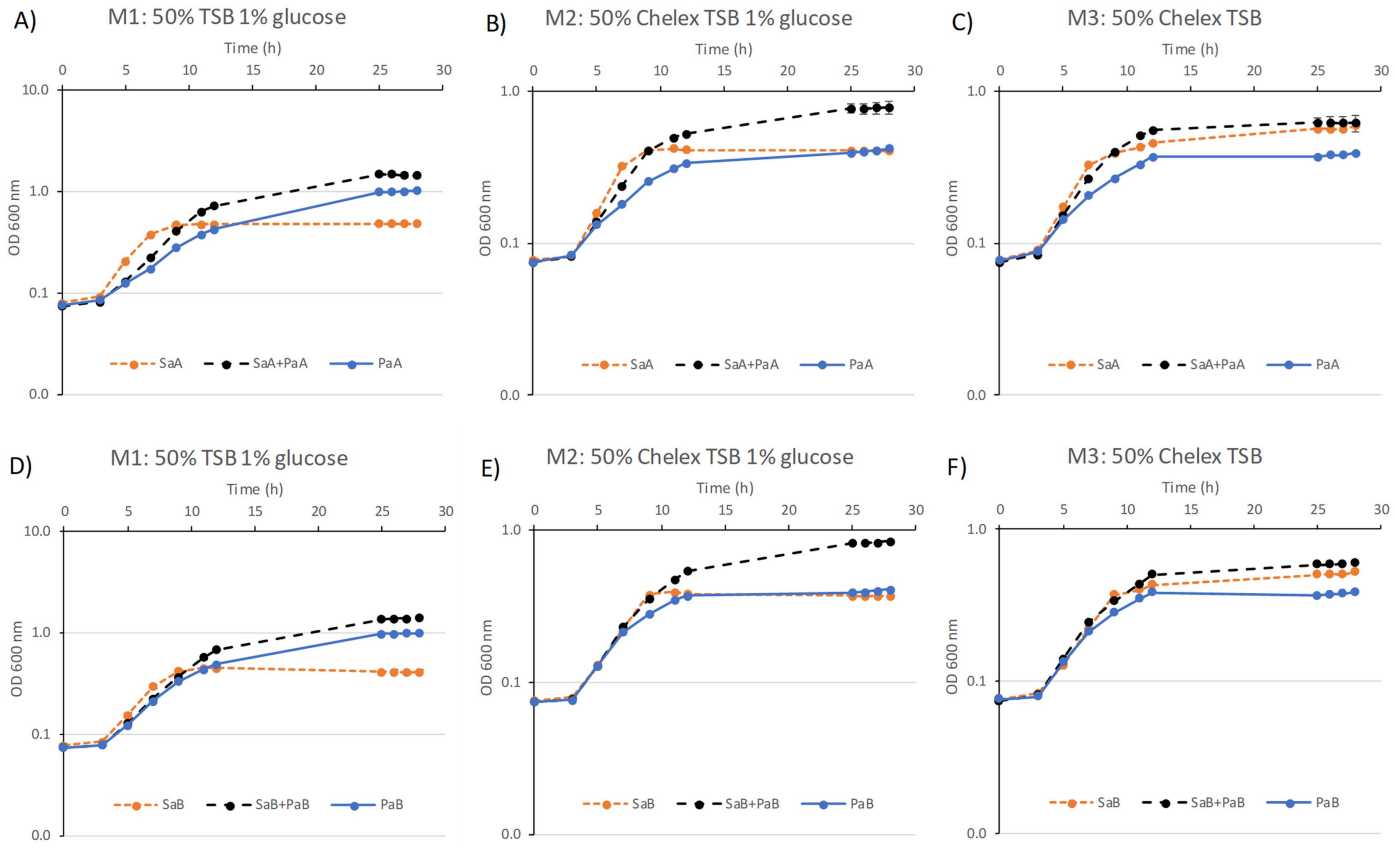
Growth curves for monocultures and cocultures of clinical strains in three different medium conditions: **(A,D)** M1, **(B,E)** M2, **(C,F)** M3 for two clinical pairs of isolates **(A–C)** pair A consisting of *S. aureus* (SaA) and *P. aeruginosa* (PaA), **(D–F)** pair B consisting of *S. aureus* (SaB) and *P. aeruginosa* (PaB). Dotted orange line represents monoculture of *S. aureus* (*Sa*), solid blue line monoculture of *P. aeruginosa* (*Pa*) and black dashed line the coculture of the two. Points represent averages of six biological cultures (error bars representing standard deviation are smaller than the data points in the graph). The culture density at the end of stationary phase is significantly different between monocultures of *Sa* and *Pa*, as well as between *Pa* and coculture and between *Sa* and coculture in all conditions (Student’s *t*-test, *p* < 0.01, *y*-axes is in graphs are logarithmic).

Three medium compositions were used: one representing standard growth conditions for airway pathogens including glucose, as previously described (M1) (Gomes-Fernandes et al., 2022), one Fe-depleted standard condition with glucose (M2) and one Fe depleted condition without glucose (M3). In all three liquid media conditions, the pairs of clinical strains grew to higher biomass density when grown as coculture than as monocultures (Figure 2).

**Figure 3:**
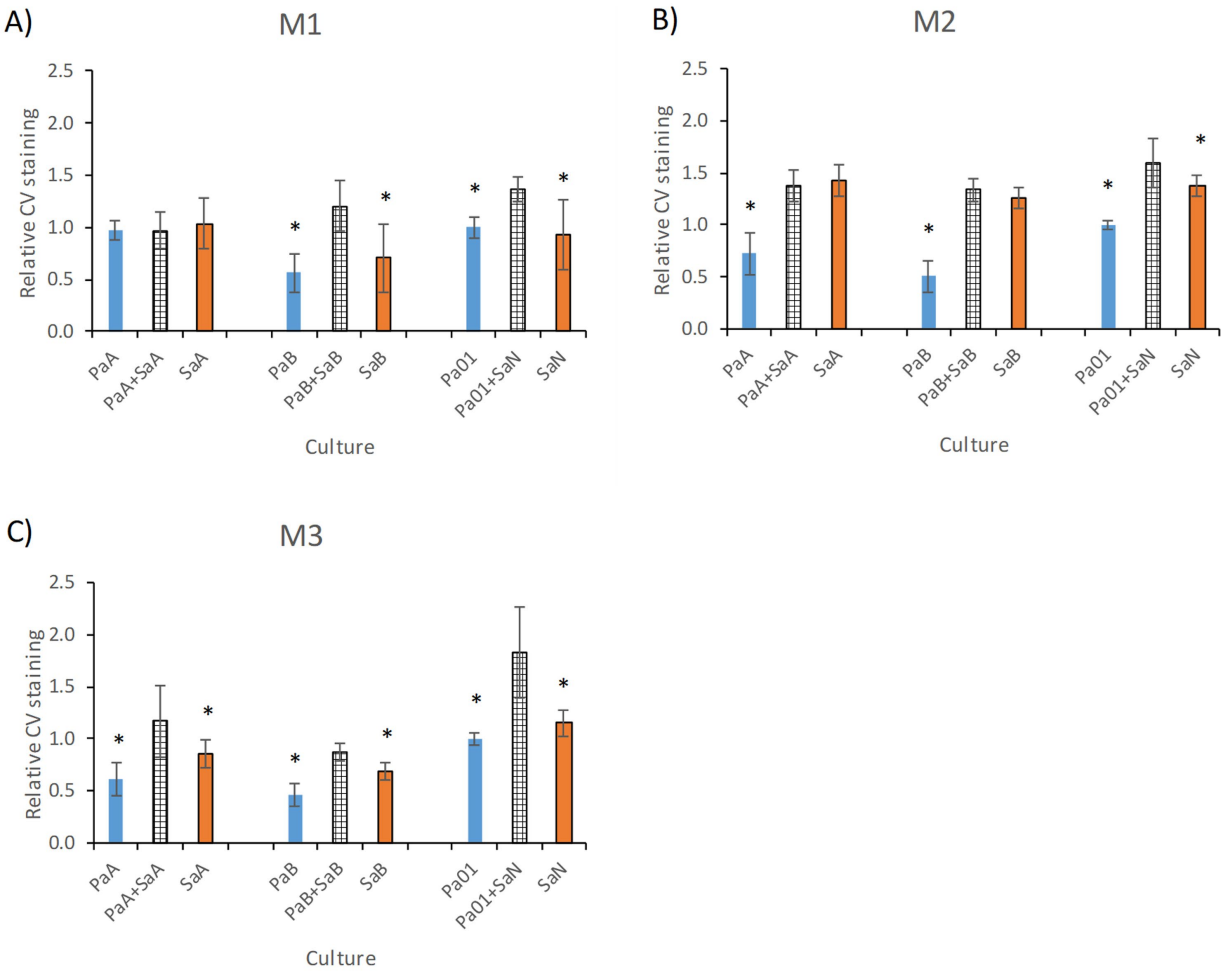
Relative biofilm formation by monocultures and cocultures in the three media: **(A)** M1, **(B)** M2, and **(C)** M3. Biofilm formation was assessed by the crystal violet assay and the biofilm volume normalized to that of the reference stain Pa01 in each medium. The bars represent an average of 12 culture wells in 96-well plates from two different biological replica performed on different days. Error bars represent standard deviation. *Represent significantly different values (*p* < 0.01, Students *t*-test) between monoculture and coculture. Blue bars represent *P. aeruginosa* (*Pa*) and red bars *S. aureus* (*Sa*), crossed bars cocultures.

The largest increase in biomass compared to monocultures was observed in the M2 medium (Fe-free medium with 1% glucose, Figures 2A,D). An increase was also seen for cultures in the M1 medium (50% TSB with 1% glucose, Figures 2B,E). In contrast, the coculture in M3 (Fe-free medium without glucose) remained at a culture density similar to what was observed for the *Sa* monoculture (Figures 2C,F). The monocultures of *Pa* grew to higher densities than *Sa* in presence of glucose (M1) and the opposite was observed in absence of glucose (M3). In line with the increase in optical density in cocultures, cultivation in biofilms also generally gave rise to a higher biomass for cocultures than for monocultures (Figure 3), with the exception for the coculture between PaA and SaA in M1 medium. Some differences were observed between optical density measurements in liquid culture and biofilms, most likely reflecting differences in biofilm-forming ability between the strains.

Growth of the clinical isolates on blood agar enabled easy distinction between colonies of *Sa* and *Pa* due to differences in colony morphology. This was observed both for the two clinical pairs as well as for the mixture of reference strains (Supplementary Figure 1). On blood-agar plates, the two clinical stains of *Pa* showed less pigmentation than the reference Pa01, as previously reported (Rzhepishevska et al., 2018). A difference in colony morphology was also observed for the two *Sa* isolates. On blood agar the *Sa* strain from pair B (SaB) grew to smaller colony size compared to the *Sa* strain from pair A (SaA). In coculture with PaB, SaB exhibited even smaller colonies indicating additional growth disadvantage when growing in close presence of *Pa* even in the nutrient rich condition of blood agar (Supplementary Figure 1). *Pa* has previously been described to release pyoverdine, pyochelin and 2-heptyl-4-hydroxyquinoline making *Sa* shift metabolism to fermentation and grow more slowly (Camus et al., 2022) which on plates gives a small colony morphology. These differences in colony morphology during growth as coculture on plates further indicated possible competition between some of the strains with respect to Fe.

**Figure 4:**
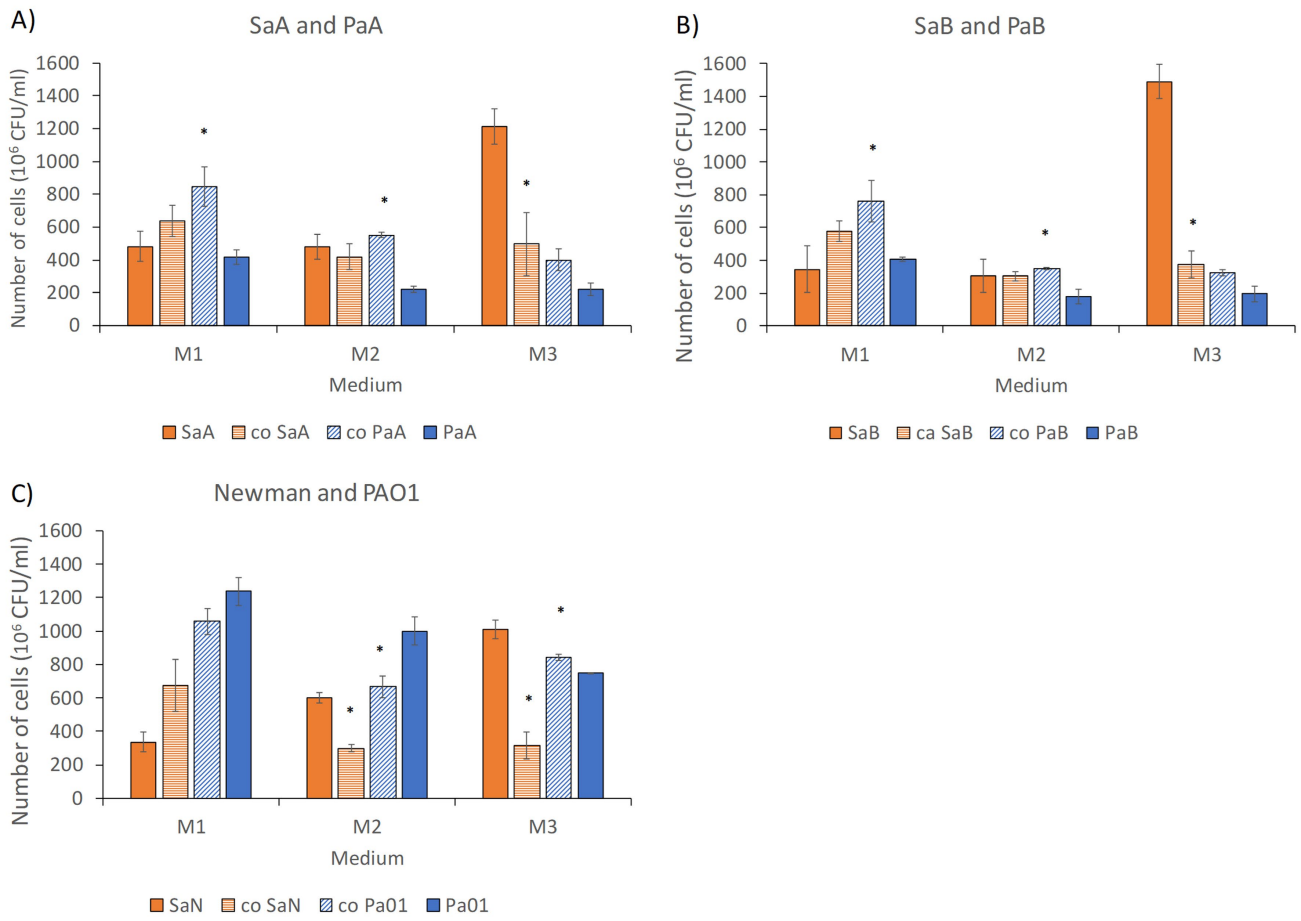
Number of viable cells (colony forming units) remaining after 15 h of monocultures or coculture of *S. aureus* (*Sa*) and *P. aeruginosa* (*Pa*) strains in three different media: M1-50% TSB with 1% glucose, M2-50% chelex-treated TSB with 1% glucose, and M3-50% chelex-treated TSB, for the couples: **(A)** SaA and PaA, **(B)** SaB and PaB, **(C)** reference strains SaN (Newman) and Pa01. Solid orange bars represent monocultures of *Sa*, striped orange bars represent *Sa* in coculture with *Pa*, striped blue bars *Pa* in coculture with *Sa*, and solid blue bars monoculture of *Pa*. The bars represent average between three plates and error bars standard deviation. Significant difference between monoculture and coculture (*t*-test, *p* < 0.01) is noted with a star.

In order to better understand the biomass variations observed in the three media, M1–M3 (Figures 2, 3) we performed viable cell counts after 15 h growth (Figure 4). The counts revealed that primarily *Pa* was benefitting from the coculture in presence of glucose (M1 and M2), shown as an increase in cell numbers for both clinical pairs (Figures 4A,B). However, the cell count of *Sa* was not significantly altered between mono- and coculture in presence of glucose. The Fe-free conditions without glucose (M3) supported growth of *Sa* to higher extent than *Pa* in monocultures. However, in M3 cocultures the number of *Sa* cells decreased significantly compared to monoculture (Figure 4), despite that the total culture density did not change to a large degree under these conditions (Figure 2). This indicated that some form of interactions took place between the species during coculture in Fe-free conditions that was not favorable for *Sa*. Similar patterns were observed in the reference pair. The *Sa* (SaN) strain grew to higher densities in monoculture than in coculture in absence of Fe (M2 and M3), whereas *Pa* (Pa01) only grew to higher densities in coculture in M3. However, the interactions for the reference strains in presence of glucose differed from what was observed for the clinical strains (Figure 4C). The reference strain Pa01 did not appear to benefit from coculture in presence of glucose in the same way as the clinical *Pa* strains did.

In summary, the growth and biofilm experiments showed that nutrient conditions affected the interactions between the two species and especially in the clinical-strain pairs. The culture density in liquid broth (Figure 2) reached higher optical densities for cocultures than monocultures in presence of glucose (M1 and M2) but this was not observed in Fe-free conditions in M3. *Pa* appeared to benefit from co-cultivation in M1 and M2. In M3 *Sa* viable counts were significantly lowered in presence of *Pa*. Biofilms from clinical cocultures reached significantly higher biomass than single cultures in M3. To investigate this further we examined how the conditions influence metabolite patterns intracellularly and extracellularly in the bacterial cultures.

### 3.3 How is medium composition affecting the bacterial metabolism?

Bacterial metabolites were investigated in two steps. Analyses were first done on filtered spent medium remaining after removal of bacteria cells in order to investigate the remaining metabolites in the media giving indications of uptake from the culture medium as well as release of secondary metabolites to the culture medium. Thereafter, the metabolite pattern inside the removed cell pellets was investigated. This stepwise approach enabled metabolite patterns in the cell pellet and supernatant to be compared from the same cultures. Of the significant metabolites monitored, some were detected in both supernatants and cell pellets, but the majority were only observed in one of the compartments.

#### 3.3.1 Supernatant metabolites

**Figure 5:**
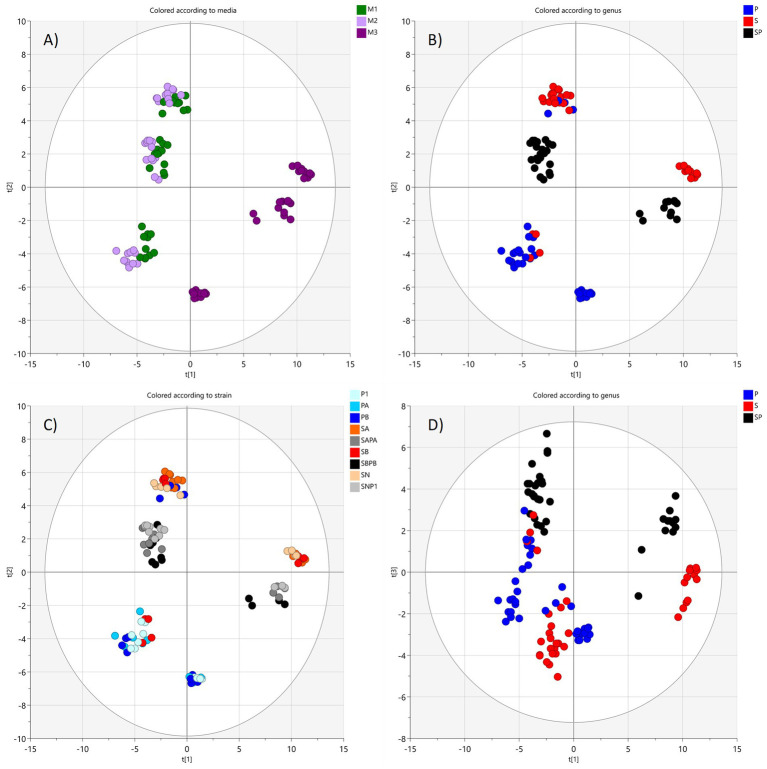
PCA score plots for supernatant samples, **(A–C)** with principal components 1 and 2, color coded according to **(A)** medium (M1 green, M2 lilac, M3 purple); **(B)** species (*P. aeruginosa* blue, *S. aureus* red, and coculture black); and **(C)** strain (different shades of blue for *Pa*, red for *Sa*, and gray for cocultures); **(D)** PCA score plot with principal components 1 and 3 color coded according to species showing cocultures at the top (black dots) and monocultures at the bottom (red and blue). Ellipse represents Hotelling’s *T*^2^ (95%) confidence region.

Principal component analysis (PCA) score plots of supernatant samples showed separation in multivariate space relating to initial medium composition (M1, M2 and M3) in the first principal component (explaining 39% of the variation) and monocultures/cocultures in the second component (*Pa* = P, *Sa* = S and cocultures = SP, 20% of the variation, Figure 5).

The third component separated monocultures and cocultures (explaining 11% of the variation). Thus, the largest differences in the supernatant data were induced by the medium composition and the second largest between bacterial species grown as monocultures and cocultures. Supernatants from media M1 and M2 were overlapping in the plot indicating close resemblance. In addition, no clear separation could be seen relating to bacterial strains from each species. Thus, the strains were grouped into species categories for continued analysis. The loading plots of the PCA showed a variety of different sugar metabolites correlated to the M1 and M2 media, confirming the influence of glucose on the metabolite pattern (Supplementary Figure 2A). PCA is a good statistical tool to reveal major trends in datasets. However, to allow for pairwise comparisons, OPLS-DA modeling and *t*-tests were applied. OPLS-DA models were, thus, created to investigate more in detail which metabolites contributed most to the significant separation between samples from media M1 and M3, for the different cultures. The weighted loadings for the OPLS-DA plots confirmed that the separation was mainly caused by various sugars or sugar metabolites remaining in the M1 medium, as well as amino acids and organic acids remaining or having been released into in the Fe-free medium (Table 1 and Supplementary Figure 3). A few metabolites were important for the separation only in monocultures. Examples of these were succinic acid, glyceric acid, and methionine. This difference indicates that the coculture metabolite pattern is not only a sum of the two species growing together but specific metabolic adaptations occurring in coculture.

#### 3.3.2 Metabolites in cell pellet

**Figure 6:**
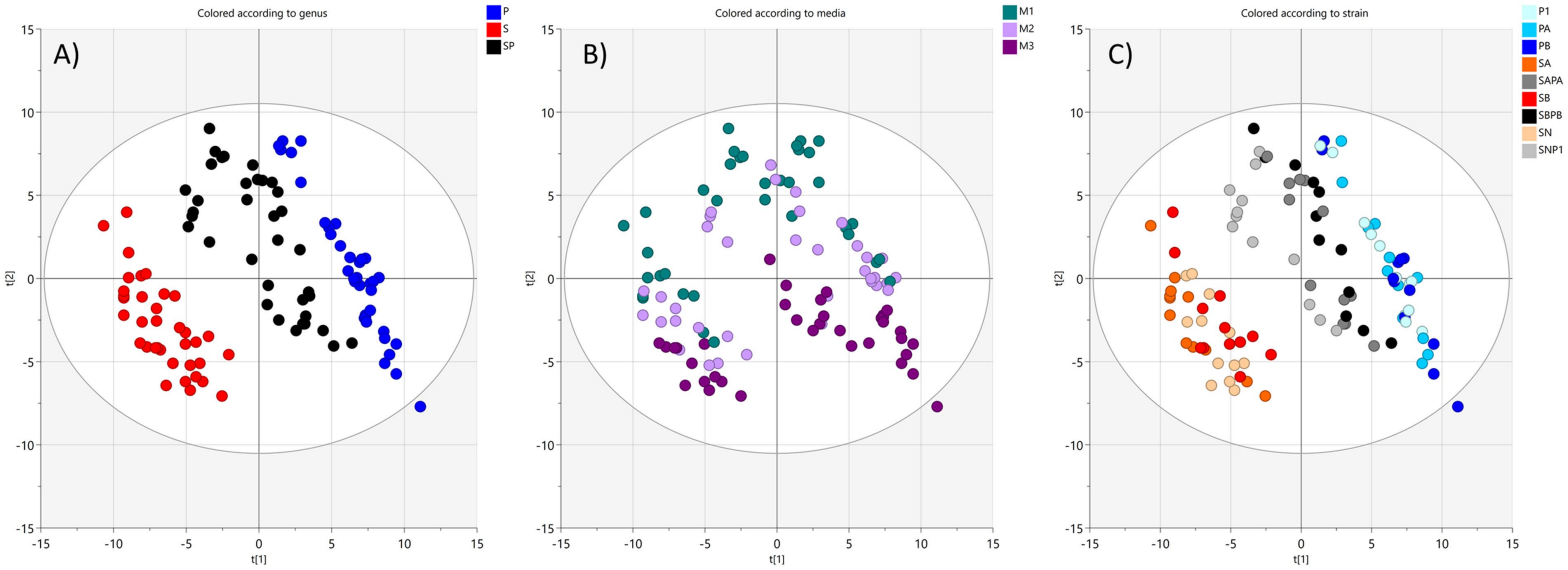
PCA score plot showing the separation of metabolite data from bacterial cell pellets in multivariate space color coded to show influence of **(A)** monoculture or coculture (red *Sa*, blue *Pa*, black coculture), **(B)** culture medium composition (M1 green, M2 lilac, M3 purple), **(C)** bacterial strain (different shades of blue for *Pa*, red for *Sa* and gray for cocultures). Ellipse represents Hotelling’s *T*^2^ (95%) confidence region. One sample is an outlier.

Unsupervised PCA analysis of the metabolite dataset from cells showed that they separated in multivariate space in a similar way as the supernatant samples did. However, for cell-pellet samples, variations linked to bacterial species gave rise to the largest spread in the multivariate space and thereafter the medium composition. The separation in the first principal component explained 30% of the variation and was related to species where the monocultures (S or P) were at either end of the scale and the cocultures (SP) in-between (Figure 6A).

The second principal component related to the culture medium where the medium with 1% glucose (M1) and the chelex-treated medium (M3) were the most separated. The chelex-treated medium with 1% glucose (M2) was in between the other two (Figure 6B, 15% of the variation). This was expected, since M2 was prepared as an intermediate between M1 and M3, and medium induced differences in metabolite profiles should be more pronounced inside cells than in the supernatant. Thus, the cell data are consistent with the supernatant data. In the PCA loading, the separation correlated with various sugar metabolites linked to metabolism in M1 and M2 (Supplementary Figure 2B) and amino acids and fatty acids for M3. Similar to the supernatant data, the cell samples from different strains were overlaid in the PCA plot, indicating metabolic similarities (Figures 5C, 6C). The lack of statistically significant difference between the metabolite patterns for the isolates from the same species was also confirmed in univariate statistical evaluation of the raw data. Therefore, metabolite patterns for strains/isolate samples were only investigated as species groups for both cells and supernatants.

Thereafter, supervised OPLS-DA analyses were performed of intracellular metabolites comparing cells cultivated in M1 and M3 (Table 2 and Supplementary Figure 4). For all cultures, in M1, uridine was increased. Uridine is a nucleoside, and its accumulation may be linked to both recycling of nucleosides or biosynthesis. The weighted loadings for *Pa* monocultures show that in the M1 medium amino acids, and sugars derivatives were increased (fructose, glycerol phosphate, glutamic acid, gluconic acid), indicating the use of sugars as a carbon sources followed by synthesis of amino acids such as glutamic acid, aspartic acid and lysine. For *Sa* monocultures, metabolites linked to sugars and glycolysis were also increased in the M1 medium (ribitol, glucose, gluconic acid derivative, glycerol phosphate, trehalose).

In Fe-free medium (M3) all cultures had an increase of metabolites such as glycerol, homoserine (a reactive version of the amino acid serine) and the saturated fatty acid myristic acid (C14). Myristic acid has been suggested to be important in membrane restructuring together with palmitic acid (Sobocińska et al., 2018) and glycerol is used by many bacterial species as a carbon source (Poblete-Castro et al., 2020) and as a building block in lipid biosynthesis. These general changes in all cultures indicated some medium-induced metabolic effects similar in all cells in absence of Fe. This appears to have induced utilization of alternative carbon sources such as glycerol and fatty acids. The increase of these two metabolites intracellularly may also indicate processes related to alterations of bacterial membrane structures to incorporate or remove saturated lipids. Both *Pa* and *Sa* have been reported to be able to use exogeneous fatty acids and incorporate them into their membranes (DeMars et al., 2020; Dubois-Brissonnet et al., 2016). In addition, lipid katabolic processes may be in place resulting in a build-up of glycerol and fatty acids in the cell (Kim and Gadd, 2019).

*Pa* monocultures in the Fe-free medium (M3), showed enrichment of fatty acids and organic acids inside the cells. These included: metabolites linked to the TCA cycle (malic acid), CoA production (beta-cyano alanine forming beta alanine), intermediates in the glyoxylate cycle using fatty acids to produce carbohydrates (oxalic acid) (Huang et al., 2018) as well as C18 fatty acid (octadecanoic acid) that may be linked to membrane re-modeling or function as a substrate for the glyoxylate pathway. Interestingly, the medium dataset (Table 1) showed presence of octadecanoic acid in the spent medium. This could indicate that the increase observed intracellularly was due to an active uptake from the medium of this fatty acid for usage in the glyoxylate cycle, rather than an accumulation resulting from lipid degradation. However, an excessive degradation could also release this fatty acid out into the supernatant, and the current data cannot conclusively reject one of these processes. Clinical isolates of *Pa* bacteria have previously been described to use fatty acids as a main carbon source when growing in the airways (Wang M. et al., 2025). This is in line with the observation of increased amounts of fatty acids intracellularly in *Pa* for the Fe-depleted medium and would indicate that the higher levels intracellularly may have been a result of active uptake.

For *Sa* monocultures in Fe-free medium (M3) an increased level of 6-hydroxynicotinic acid was observed. This may be a metabolite of nicotinic acid, a vitamin B3 variant (vitamer) that previously has been described to enable *Sa* to grow well in minimal medium (Knight, 1937). In addition, there was an increase in amino acids (beta-alanine, phenylalanine, lysine, arginine, beta-cyano alanine).

The coculture data showed similar metabolites driving the separation between M1 and M3, as in the monocultures. However, metabolites such as cystathionine, adenosyl-methionine and ribose appeared to have increased in cocultures indicating build-up of metabolites linked to cysteine and/or methionine synthesis and nucleic acid metabolism. In coculture the vitamer accumulating inside *Sa* was no longer seen, possibly indicating that this metabolite was instead consumed or that the *Sa* metabolism was redirected and this substance was no longer accumulated in the cells. Intracellular succinate and malic acid in cocultures may suggest that co-cultures utilized a broader range of carbon sources, as has previously been described for cocultures of *E. coli* and *Pa* (Yasir et al., 2024).

In summary, the differences in metabolite profiles indicated clear differences in cell metabolism between the two medium conditions. In medium M1, the metabolism was dependent on sugars as a carbon source for all cultures. This finding is not surprising as the medium contained a large number of these types of substrates. In the Fe-free medium, M3, fatty acids and organic acids appear to have been the main carbon sources. Furthermore, the metabolite patterns in cocultures indicated that the lower nutrient content gave rise to a wider range of carbon sources being used in cocultures than in monocultures, in the M3 medium. These observations could indicate both that the strains are “sharing goods” in the form of secondary metabolites or be a result of redirection of metabolic pathways during Fe-limited growth conditions limiting competition and utilizing resources differently (Culp and Goodman, 2023; Evans et al., 2020).

## 4 Discussion

This study contributes to the ongoing research effort trying to understand the interactions between *Sa* and *Pa* during co-infection. Here we focus on clinical isolates that have sequentially colonized intubated patients in an intensive care unit. For both pairs, the second colonizer, *Pa*, had been previously shown to be a weak colonizer in monoculture. We hypothesized that the sequentially colonization of patients under mechanical ventilation were related to (1) surface preconditioning performed by *Sa* facilitating the later arrival of the weaker colonizer *Pa,* and/or (2) metabolic interactions between the two species. In contrast with many other studies, we have focused on clinical isolates from intensive care and for patients without cystic fibrosis. The cystic fibrosis disease state gives rise to a very specific type of environment in the lungs and drives to specific selection pressures on bacteria. Thus, other scenarios and isolates are important to study in order to obtain a broad understanding of how pathogens in the airways interact.

The first hypothesis related to surface preconditioning. Ventilators are constructed from polymeric materials and thus, in this study hydrophobic culture plastic was used as a proxy for ventilator surfaces. Hydrophobic materials are prone to become preconditioned which may enhance bacterial attachment (Ramstedt et al., 2019; Tuson and Weibel, 2013). However, we did not find that preconditioning of surfaces with substances secreted by *Sa* altered the colonization of *Pa* significantly (Figure 1). Thus, these results suggest that the weak colonization is not improved by surface preconditioning by the *Sa* strains. In other words; *Sa* is not “paving the way” for *Pa*. This suggested that other parameters than surface physicochemical properties were at play in the sequential colonization.

The second hypothesis was based on *Pa* acquiring metabolic advantages from coculture via competition or “sharing of goods.” To investigate this, the growth of the isolates was monitored in monocultures as well as cocultures in different nutrient conditions. Cocultures grew to higher cell densities than monocultures in presence of glucose and similar densities in Fe-free conditions. This growth was in line with what has previously been described for in nutrient rich conditions, where *Pa* has been described to grow to higher culture densities (Gomes-Fernandes et al., 2022). The cocultures appeared to be beneficial for *Pa* under all conditions investigated. However, coculture appeared detrimental to *Sa* in Fe-limited conditions. This was in line with previous finding showing strain specific out-competition of *Sa* by *Pa* during nutrient limitation (Nguyen et al., 2016). Gomes-Fernandes explained this as increased quorum sensing in *Pa* giving rise to antagonistic behavior in presence of *Sa* cells, which was not induced only by culture supernatants but required presence of cells (Gomes-Fernandes et al., 2022). It has also described that *Pa* can force *Sa* to shift to fermentative metabolism and make use of organic acids and other metabolites produced such as lactate and acetoin (Yung et al., 2021). Similar effects were observed in our cocultures of VAP isolates where for example succinate was taken up from the medium and found intracellularly during growth in Fe-free conditions. However, in the glucose rich medium both monocultures and cocultures appeared to have utilized various sugars and sugar metabolites as carbon sources. Thus, in media with ample supply of glucose this shift to fermentative metabolism may not occur in the same way, or at all.

The results presented in this study suggests that interactions between species during co-infections in the airways may be complex and vary depending on the specific conditions occurring in the microenvironment around the ventilator where the bacteria reside. The *in vitro* data suggests that that presence of *Sa* biofilm may facilitate colonization of *Pa* because it provides additional nutrients. In Fe-free conditions, expected in the airways, interactions with *Pa* would be less favorable for *Sa*. Thus, it is likely that during co-infections with *Pa*, *Sa* would be outcompeted or diminished as was observed in the two patients from where the isolates originated. This may be due to several factors, *Pa* may sequester Fe from the surroundings more efficiently and/or shift the metabolism of its neighbor to fermentative pathways, as previously described (Filkins et al., 2015). Thus, the bronchial colonization record of the patients from which the strains were originally isolated, indicate nutrient limited conditions in the airway fluids tilting the balance between the two species in favor of *Pa*. These findings are of clinical interest since changes may occur in the human airway fluid during disease that alter the availability of key nutrients such as glucose and iron. Thus, in the airways the concentration of glucose may differ between patients. It has been described to be 0.4 mM in healthy individuals but can increase due to different disease states such as diabetes, obstructive pulmonary disease and cystic fibrosis (Baker and Baines, 2018; Mallia et al., 2018; Gill et al., 2016; Vasiljevs et al., 2023). Increased glucose levels in the airway fluids have been described to result in higher bacterial loads (Baker and Baines, 2018; Mallia et al., 2018; Gill et al., 2016). In line with what was observed here *in vitro*. These increases probably are a result of these conditions benefitting many types of bacterial species and not inducing competitive behaviors between species. The other factor investigated here, iron, is an essential nutrient for humans but is tightly regulated in the body for example by proteins chelating iron, in order to avoid redox induced damages to cells and tissues but also to limit pathogen growth. This is also the case in the airways where iron is present but tightly regulated for example by iron binding proteins such as lactoferrin (Zhang et al., 2019; Neves et al., 2019). However, several disease states have been reported to disrupt iron homeostasis in the lungs which may lead to increased availability to pathogens (Neves et al., 2019). This study shows that the interactions between bacteria are altered during these different conditions and indicates that increased levels of glucose and/or Fe in airways likely lowers the competition between bacteria such as *Pa* and *Sa* and may give rise to overall higher bacterial load. Thus, the nutrient conditions present in each patient at the site of infection will affect interactions between pathogens and influence the level of competition. The limitation of the study is that it is a case study based on two sequentially isolated pairs of *Sa* and *Pa*. The data-driven approach used serves to provide a foundation for developing hypotheses that later need to be tested in other larger or more mechanistically focused studies. Consequently, the study should be followed by future more detailed investigations of mechanisms giving rise to the observed metabolite patterns. The data indicate that the interactions between bacterial strains may differ from patient to patient, not only due to differences in bacterial strains but also due to host factors at the site of infection. This hypothesis, also needs to be tested in larger studies by recording relevant patient data that may influence the bacterial interactions taking place during an infection. Based on this study factors such as glucose and Fe would be important to monitor in such a follow-up study.

## Data Availability

The original contributions presented in the study are included in the article/Supplementary material, further inquiries can be directed to the corresponding author.

## Footnotes

1
https://github.com/CreMoProduction/nistAutoSearch
