# Supplementary material for: Paving the way or sharing goods?—Interactions between pairs of Staphylococcus aureus and Pseudomonas aeruginosa sequentially isolated from respiratory samples of patients on mechanical ventilation
Source: Front Microbiol. 2026 Apr 15;17:1798383. doi: 10.3389/fmicb.2026.1798383 (PMC13127463; doi:10.3389/fmicb.2026.1798383)
Supplement: Supplementary file 1 [file Data_Sheet_1.pdf]

Supplementary Material for

Paving the Way or Sharing Goods? –Interactions between pairs of *Staphylococcus aureus* and *Pseudomonas aeruginosa* sequentially isolated from respiratory samples of patients on mechanical ventilation

John Erlingsen[1], Dmytro Sokol[1], Oleksandr Ilchenko[1], Meissiner Gomes-Fernandes[2,3], Olena Rzhepishevska[1], Cristina Prat-Aymerich[2,3,4], Henrik Antti[1], Alicia Lacoma[2,3], Madeleine Ramstedt[1]*

[1]Department of Chemistry, Umeå Centre of Microbial Research, Umeå University, 901 87 Umeå, Sweden

[2]Microbiology Department, Hospital Universitari Germans Trias i Pujol, Institut d'Investigació en Ciències de la Salut Germans Trias i Pujol, Universitat Autònoma de Barcelona (UAB), Badalona, Spain

[3]CIBER Enfermedades Respiratorias, CIBER, Instituto de Salud Carlos III, Badalona, Spain.

[4]Julius Center for Health Sciences and Primary Care, University Medical Center Utrecht, Utrecht University, Utrecht, The Netherlands

*** Correspondence:**
Corresponding Author
madeleine.ramstedt@umu.se

1 Supplementary Figures and Tables

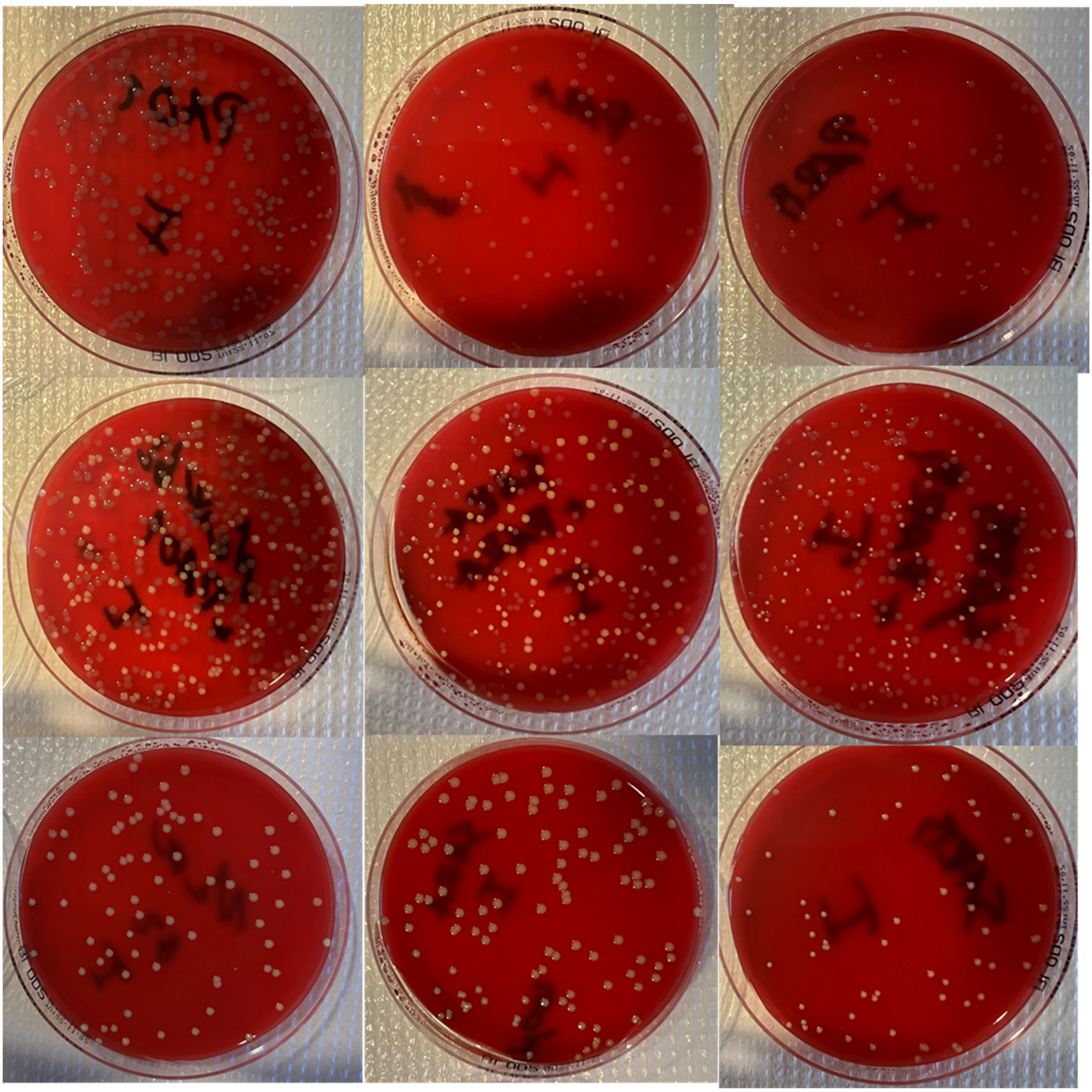

Supplementary Figure 1. Photos of colony morphologies for bacterial strains grown on blood agar plates from cultures in the M1 medium (50% TSB with 1% glucose). Top row represents *Pa* strains, middle row cocultures, and bottom row *Sa*. First column represents monoculture of reference strains Pa01 at the top, the coculture Pa01 and SaN in the middle and monoculture of SaN at the bottom. The second column represents PaA, PaA with SaA, and SaA. The third column PaB, PaB with SaB, and SaB.

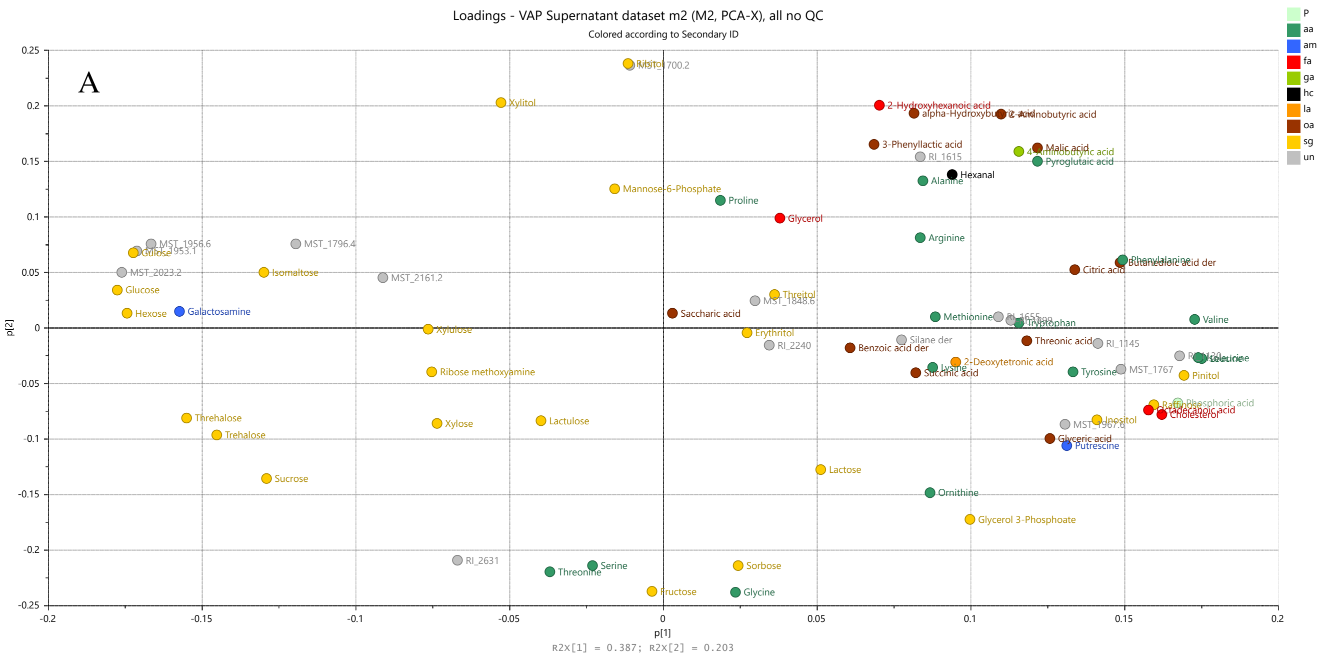
Loadings - VAP Supernatant dataset m2 (M2, PCA-X), all no QC
Colored according to Secondary ID
A
P
aa
am
fa
ga
hc
la
oa
sg
un
p[1]
p[2]
R2X[1] = 0.387; R2X[2] = 0.203
Xylitol
2-Hydroxyhexanoic acid
alpha-Hydroxybutyric acid
2-Aminobutyric acid
3-Phenyllactic acid
RI_1615
Malic acid
4-Aminobutyric acid
Pyroglutaic acid
Hexanal
Alanine
Mannose-6-Phosphate
Proline
Glycerol
Arginine
MST_1956.6
MST_1796.4
MST_2023.2
Isomaltose
MST_2161.2
Glucose
Hexose
Galactosamine
Citric acid
Butanedioic acid der
Threitol
MST_1848.6
Saccharic acid
Methionine
Tryptophan
Valine
Xylulose
Erythritol
RI_2240
Silane der
Benzoic acid der
Threonic acid
RI_1145
2-Deoxytetronic acid
Succinic acid
Tyrosine
MST_1767
Pinitol
Ribose methoxyamine
Phosphoric acid
Threhalose
Trehalose
Xylose
Lactulose
Inositol
Octadecanoic acid
Cholesterol
Glyceric acid
Putrescine
Sucrose
Lactose
Ornithine
Glycerol 3-Phosphoate
RI_2631
Serine
Threonine
Sorbose
Fructose
Glycine

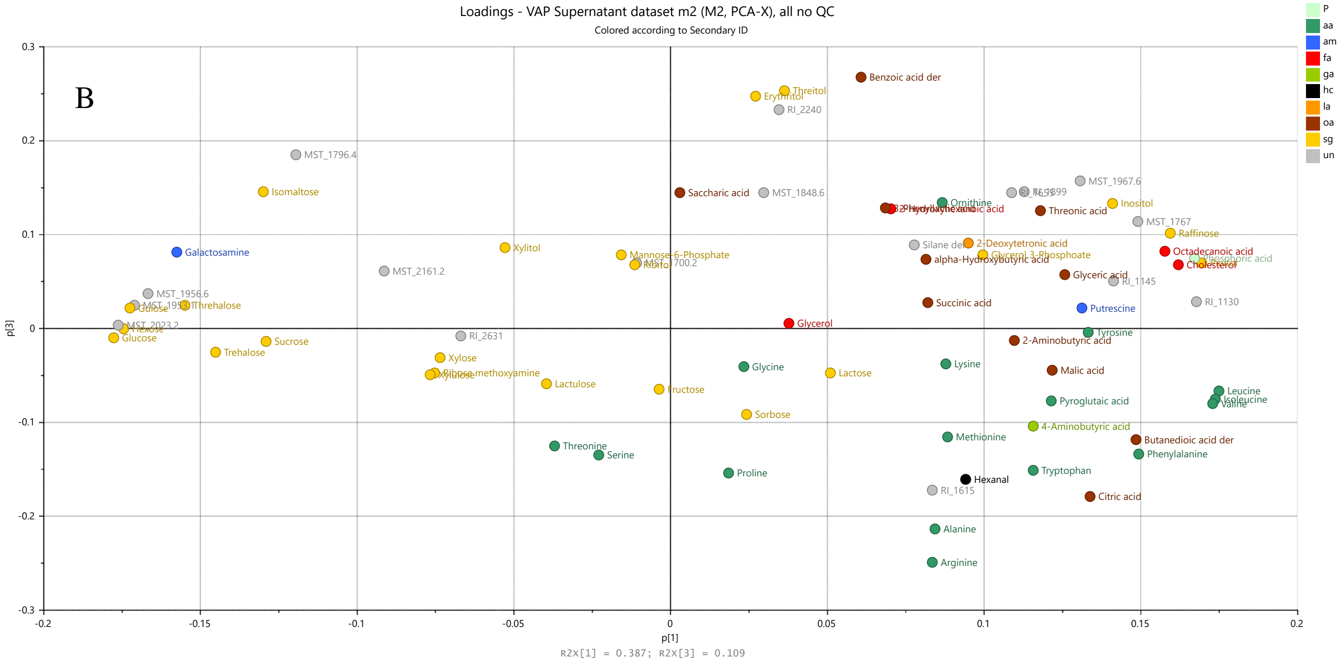
Loadings - VAP Supernatant dataset m2 (M2, PCA-X), all no QC
Colored according to Secondary ID
B
P
aa
am
fa
ga
hc
la
oa
sg
un
p[1]
p[3]
R2X[1] = 0.387; R2X[3] = 0.109
Benzoic acid der
Erythritol
Threitol
RI_2240
MST_1796.4
MST_1967.6
Isomaltose
Saccharic acid
MST_1848.6
Ornithine
Inositol
Threonic acid
MST_1767
Raffinose
Galactosamine
Xylitol
Mannose-6-Phosphate
MST_2161.2
2-Deoxytetronic acid
Glycerol 3-Phosphoate
alpha-Hydroxybutyric acid
Octadecanoic acid
Phosphoric acid
Cholesterol
MST_1956.6
Threhalose
Glyceric acid
RI_1145
Succinic acid
RI_1130
Putrescine
MST_2023.2
Hexose
Glucose
Glycerol
Tyrosine
RI_2631
Sucrose
Trehalose
2-Aminobutyric acid
Xylose
Ribose methoxyamine
Glycine
Lactose
Lysine
Malic acid
Lactulose
Fructose
Pyroglutaic acid
Leucine
Isoleucine
Valine
Sorbose
4-Aminobutyric acid
Methionine
Threonine
Serine
Butanedioic acid der
Phenylalanine
Proline
Tryptophan
Hexanal
RI_1615
Citric acid
Alanine
Arginine

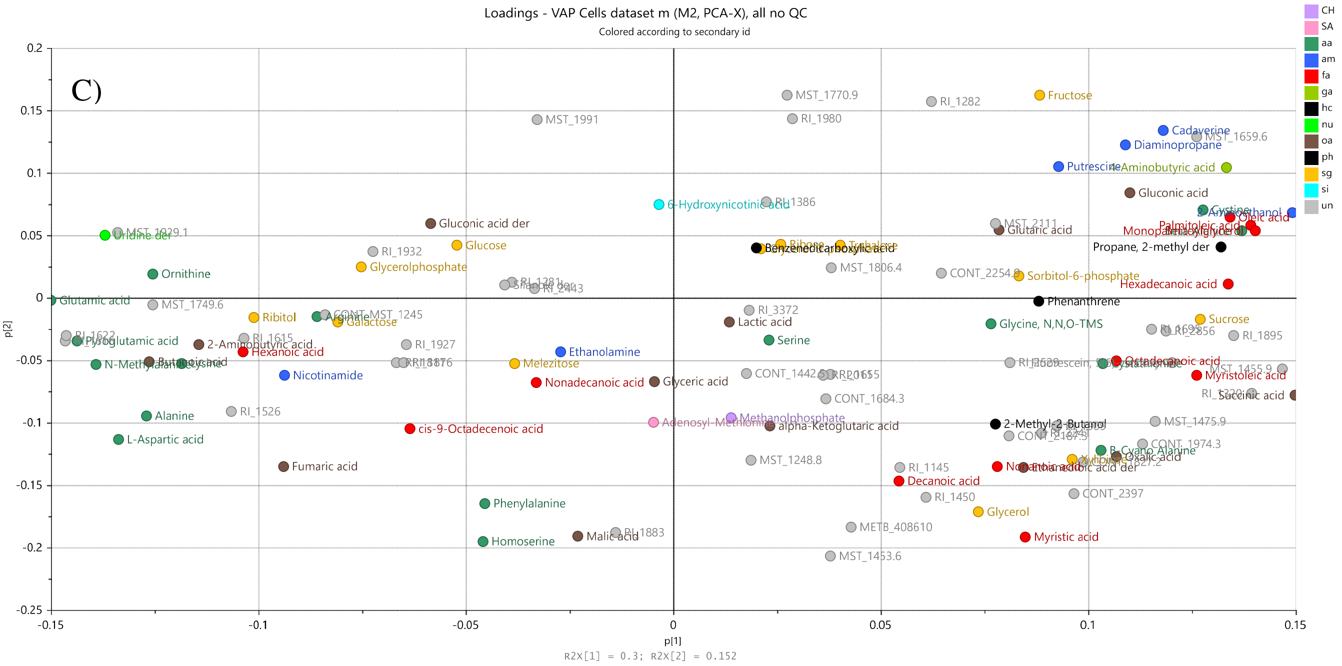

Supplementary Figure 2. Loading plots for PCA plot in a) supernatant samples in Figure 5a-c; b) supernatant samples in Figure 5d; and c) cell-pellet samples in Figure 6.

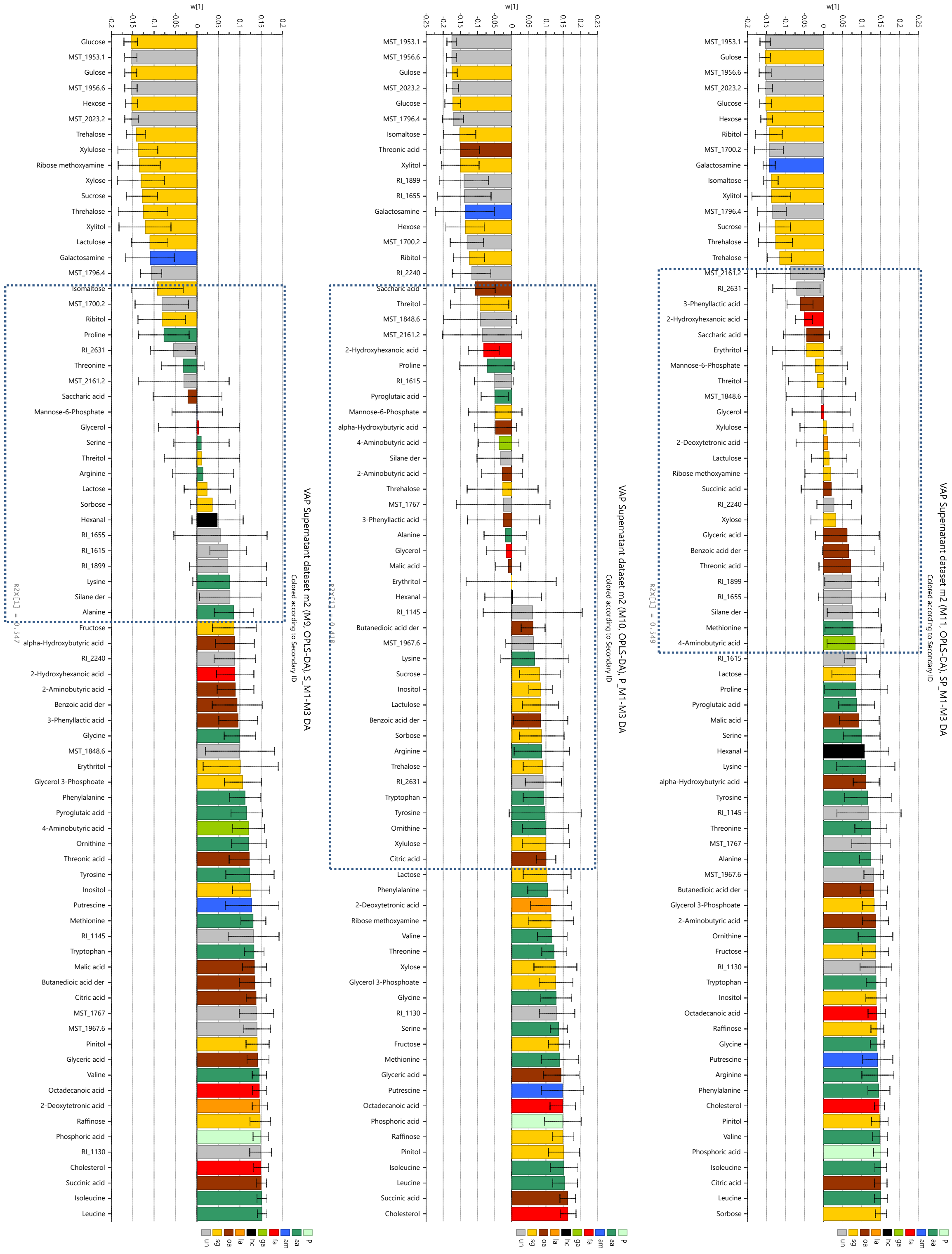
VAP Supernatant dataset m2 (M9, OPLS-DA), S_M1-M3 DA
Colored according to Secondary ID
R2X[1] = 0.547
w[1]
-0.2 -0.15 -0.1 -0.05 0 0.05 0.1 0.15 0.2
Glucose
MST_1953.1
Gulose
MST_1956.6
Hexose
MST_2023.2
Trehalose
Xylulose
Ribose methoxyamine
Xylose
Sucrose
Threhalose
Xylitol
Lactulose
Galactosamine
MST_1796.4
Isomaltose
MST_1700.2
Ribitol
Proline
RI_2631
Threonine
MST_2161.2
Saccharic acid
Mannose-6-Phosphate
Glycerol
Serine
Threitol
Arginine
Lactose
Sorbose
Hexanal
RI_1655
RI_1615
RI_1899
Lysine
Silane der
Alanine
Fructose
alpha-Hydroxybutyric acid
RI_2240
2-Hydroxyhexanoic acid
2-Aminobutyric acid
Benzoic acid der
3-Phenyllactic acid
Glycine
MST_1848.6
Erythritol
Glycerol 3-Phosphoate
Phenylalanine
Pyroglutaic acid
4-Aminobutyric acid
Ornithine
Threonic acid
Tyrosine
Inositol
Putrescine
Methionine
RI_1145
Tryptophan
Malic acid
Butanedioic acid der
Citric acid
MST_1767
MST_1967.6
Pinitol
Glyceric acid
Valine
Octadecanoic acid
2-Deoxytetronic acid
Raffinose
Phosphoric acid
RI_1130
Cholesterol
Succinic acid
Isoleucine
Leucine
un sg oa la hc ga fa am aa P
VAP Supernatant dataset m2 (M10, OPLS-DA), P_M1-M3 DA
Colored according to Secondary ID
w[1]
-0.25 -0.2 -0.15 -0.1 -0.05 0 0.05 0.1 0.15 0.2 0.25
MST_1953.1
MST_1956.6
Gulose
MST_2023.2
Glucose
MST_1796.4
Isomaltose
Threonic acid
Xylitol
RI_1899
RI_1655
Galactosamine
Hexose
MST_1700.2
Ribitol
RI_2240
Saccharic acid
Threitol
MST_1848.6
MST_2161.2
2-Hydroxyhexanoic acid
Proline
RI_1615
Pyroglutaic acid
Mannose-6-Phosphate
alpha-Hydroxybutyric acid
4-Aminobutyric acid
Silane der
2-Aminobutyric acid
Threhalose
MST_1767
3-Phenyllactic acid
Alanine
Glycerol
Malic acid
Erythritol
Hexanal
RI_1145
Butanedioic acid der
MST_1967.6
Lysine
Sucrose
Inositol
Lactulose
Benzoic acid der
Sorbose
Arginine
Trehalose
RI_2631
Tryptophan
Tyrosine
Ornithine
Xylulose
Citric acid
Lactose
Phenylalanine
2-Deoxytetronic acid
Ribose methoxyamine
Valine
Threonine
Xylose
Glycerol 3-Phosphoate
Glycine
RI_1130
Serine
Fructose
Methionine
Glyceric acid
Putrescine
Octadecanoic acid
Phosphoric acid
Raffinose
Pinitol
Isoleucine
Leucine
Succinic acid
Cholesterol
un sg oa la hc ga fa am aa P
VAP Supernatant dataset m2 (M11, OPLS-DA), SP_M1-M3 DA
Colored according to Secondary ID
R2X[1] = 0.549
w[1]
-0.2 -0.15 -0.1 -0.05 0 0.05 0.1 0.15 0.2 0.25
MST_1953.1
Gulose
MST_1956.6
MST_2023.2
Glucose
Hexose
Ribitol
MST_1700.2
Galactosamine
Isomaltose
Xylitol
MST_1796.4
Sucrose
Threhalose
Trehalose
MST_2161.2
RI_2631
3-Phenyllactic acid
2-Hydroxyhexanoic acid
Saccharic acid
Erythritol
Mannose-6-Phosphate
Threitol
MST_1848.6
Glycerol
Xylulose
2-Deoxytetronic acid
Lactulose
Ribose methoxyamine
Succinic acid
RI_2240
Xylose
Glyceric acid
Benzoic acid der
Threonic acid
RI_1899
RI_1655
Silane der
Methionine
4-Aminobutyric acid
RI_1615
Lactose
Proline
Pyroglutaic acid
Malic acid
Serine
Hexanal
Lysine
alpha-Aminobutyric acid
Tyrosine
RI_1145
Threonine
MST_1767
Alanine
MST_1967.6
Butanedioic acid der
Glycerol 3-Phosphoate
2-Aminobutyric acid
Ornithine
Fructose
RI_1130
Tryptophan
Inositol
Octadecanoic acid
Raffinose
Glycine
Putrescine
Arginine
Phenylalanine
Cholesterol
Pinitol
Valine
Phosphoric acid
Isoleucine
Citric acid
Leucine
Sorbose
un sg oa la hc ga fa am aa P

Supplementary Figure 3, weighted loadings for supernatant metabolites from monocultures of (from the left) *Sa, Pa* and cocultures. Metabolites outside the dashed box have a significance level of $p<0.01$ and are also shown in Supplementary Table 3. Metabolites high in M1 at the top and M3 at the bottom. (Colour coded bars according to subgroups: purple CH=Methanolphosphate, pink SA=Adenosyl-Methionine, green aa= amino acid, blue am= amine, red fa= fatty acid, light green ga= gaba, black hc= hydrocharbon, turquoise nu= nucleotide, brown oa=organic acid, black ph= phthalate, yellow sg= sugars or sugar derivates, light blue si= vitamin metabolite, grey un= unidentified metabolite).

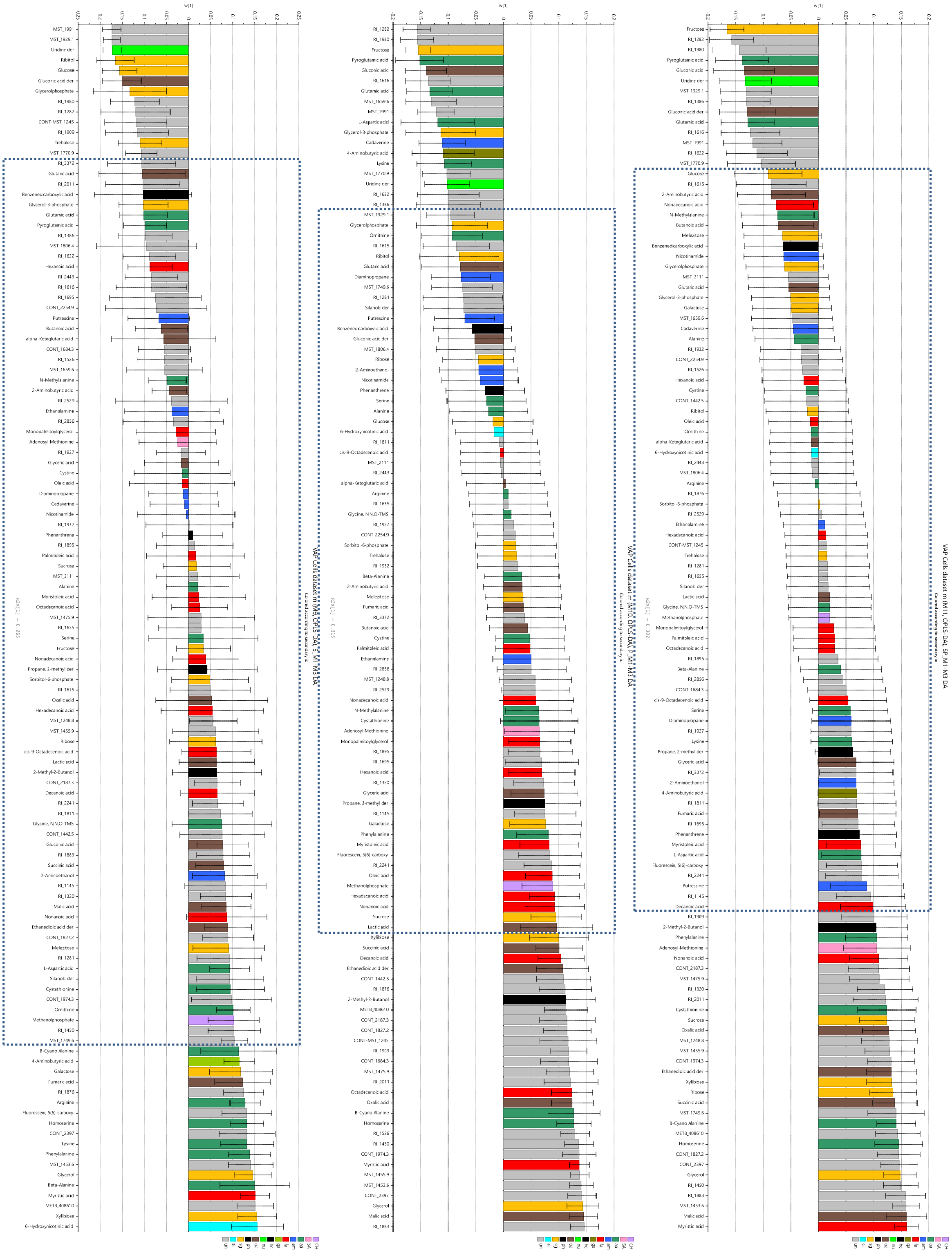

Supplementary Figure 4. OPLS-DA weighted loadings for monocultures of (from the left) *Sa* and *Pa* as well as the coculture. Cell-pellet metabolites outside the dashed box have a significance level of $p<0.01$ and are also shown in Supplementary Table 4. Metabolites high in M1 at the top and M3 at the bottom. Chelex medium to the bottom and glucose to the top. (Colour coded bars according to subgroups: purple CH=Methanolphosphate, pink SA=Adenosyl-Methionine, green aa= amino acid, blue am= amine, red fa= fatty acid, light green ga= gaba, black hc= hydrocharbon, turquoise nu= nucleotide, brown oa=organic acid, black ph= phthalate, yellow sg= sugars or sugar derivates, light blue si= vitamin metabolite, grey un= unidentified metabolite).

1.1 Supplementary Tables

Supplementary Table 1. Clinical data for Patient A and Patient B.

**Clinical patient**	**Patient A**	**Patient B**
Study Group	Tracheobronchitis	Pneumonia
Origin	Nosocomial	Nosocomial
Sex	Male	Male
Age	18	58
Reason for intensive care unit (ICU)_admission	Major trauma	Medical
Total days mechanical ventilation (MV)	38	33
Persistent isolation respiratory sample 7days *	No	Yes
Days mechanical ventilation until first isolation	7	3
Mixed bacterial isolation in the first isolate	No	No
Pseudomonas isolation after *S.aureus*	Yes	Yes
Previous MRSA Carriage#	No	No
Blood culture result	Negative	Negative
Presence of comorbidities	No	Yes
Diabetes	No	No
Chronic respiratory disease	No	No
Neoplasia	No	No
Arterial hypertension	No	Yes
Cardiac failure	No	No
Renal failure	No	No
Obesity	No	No
Immunosuppressed	No	No
Central nervous system involvement	Yes	Yes
Ischemia	No	No
Bleeding	No	Yes
Traumatic brain injury	Yes	No
X-Ray findings	No infiltrate	Diffuse
Septic shock (Respiratory focus)	No	No
Complications related to respiratory infection	No	No
Mortality	No	No
Previous antimicrobial treatment		Ciprofloxacin
Previous antimicrobial treatment	Amoxicillin clavulanate	Quinolone
Antimicrobial treatment after culture result	Cloxacillin	Linezolid
Clinical evolution	Respiratory infection cured	Respiratory infection cured

*persistence, was defined as repeated isolation of *S. aureus* in respiratory sample, despite patient was receiving antimicrobial treatment adjusted to antimicrobial susceptibility testing results. (# next page)
MRSA: Methicillin resistant *Staphylococcus aureus*

Supplementary Table 2. Antibiotic susceptibility profile for *S.aureus* and *P.aeruginosa* isolates

Antibiotic susceptibility profile	SaA
Penicillin	R
Oxacillin	S
Cefotaxin	S
Gentamicin	S
Ciprofloxacin	S
Eritromicin	S
Rifampicin	S
Clindamycin	S
Vancomycin	S
Teicoplanin	S
Trimetroprim/Sulfametoxazol	S
Mupirocin	S

Antibiotic susceptibility profile	SaB
Penicillin	R
Oxacillin	S
Cefotaxin	S
Gentamicin	S
Ciprofloxacin	S
Eritromicin	S
Rifampicin	S
Clindamycin	S
Vancomycin	S
Teicoplanin	S
Trimetroprim/Sulfametoxazol	S
Mupirocin	S

Antibiotic susceptibility profile	PaA
Piperacillin -Tazobactam	I
Ceftazidim	I
Cefepime	I
Aztreonam	S
Imipenem	R
Meropenem	R
Gentamicin	R
Amikacin	S
Tobramicin	R
Netilmicin	S
Ciprofloxacin	R
Trimetroprim/Sulfametoxazol	R
Colistin	S

Antibiotic susceptibility profile	PaB
Piperacillin -Tazobactam	I
Ceftazidim	S
Cefepime	S
Aztreonam	S
Imipenem	R
Meropenem	R
Gentamicin	R
Amikacin	S
Tobramicin	R
Netilmicin	I
Ciprofloxacin	R
Trimetroprim/Sulfametoxazol	R
Colistin	S

*) R= resistant, S= sensitive, I= intermediate, antibiotics in red text were tested on both sprecies.

Supplementary Table 3. Supernatant metabolites driving separation in OPLS-DA models in alphabetic order (weighted loadings p<0.01) comparing M3 and M1 medium for three types of cultures. Positive loadings linked to M3 and negative loadings to M1.

**Metabolite**	***Sa***	***Pa***	***Sa+Pa***
2-Aminobutyric acid	0.09		0.14
2-Deoxytetronic acid	0.15	0.12	
2-Hydroxyhexanoic acid	0.09		
3-Phenyllactic acid	0.10		
**4-Aminobutyric acid**	0.12		
Alanine			0.13
alpha-Hydroxybutyric acid	0.09		0.11
**Arginine**			0.14
Benzoic acid der	0.09		
Butanedioic acid der	0.14		0.13
Cholesterol	0.15	0.16	0.15
Citric acid	0.14		0.15
Erythritol	0.10		
Fructose		0.14	0.14
Glyceric acid	0.14	0.14	
Glycerol 3-Phosphoate	0.11	0.13	0.13
Glycine	0.10	0.13	0.14
Hexanal			0.11
Inositol	0.13		0.14
Isoleucine	0.15	0.15	0.15
Lactose			0.08
Leucine	0.15	0.16	0.15
**Lysine**			0.11
**Malic acid**	0.13		0.09
Methionine	0.13	0.14	
**Octadecanoic acid**	0.15	0.15	0.14
Ornithine	0.12		0.14
**Phenylalanine**	0.11	0.10	0.15
Phosphoric acid	0.15	0.15	0.15
Pinitol	0.14	0.15	0.15
Proline			0.09
Putrescine	0.13	0.15	0.14
Pyroglutaic acid	0.12		0.09
Raffinose	0.15	0.15	0.14
**Ribose**		0.12	
Serine		0.14	0.10

**Metabolite**	***Sa***	***Pa***	***Pa+Sa***
Galactosamine	-0.11	-0.14	-0.14
**Glucose**	-0.15	-0.17	-0.15
Gulose	-0.15	-0.17	-0.15
Hexose	-0.15	-0.14	-0.15
Isomaltose	-0.09	-0.15	-0.14
Lactulose	-0.11		
Ribitol		-0.12	-0.14
Ribose	-0.13		
Saccharic acid		-0.11	
Sucrose	-0.13		
Sucrose			-0.13
Threhalose	-0.13		-0.12
Threonic acid		-0.15	
**Trehalose**	-0.14		-0.13
Unknown (MST_1700.2)		-0.13	-0.14
Unknown (MST_1796.4)	-0.11	-0.17	-0.14
Unknown (MST_1953.1)	-0.15	-0.18	-0.15
Unknown (MST_1956.6)	-0.15	-0.18	-0.15
Unknown (MST_2023.2)	-0.15	-0.17	-0.15
Unknown (MST_2161.2)			-0.09
Unknown (RI_1655)		-0.14	
Unknown (RI_1899)		-0.14	
Unknown (RI_2240)		-0.12	
Xylitol	-0.12	-0.15	-0.14
Xylose	-0.13		
Xylulose	-0.14		

Sorbose			0.15
**Succinic acid**	0.15	0.16	
Threonic acid	0.12		
Threonine		0.12	0.12
Tryptophan	0.13		0.14
Tyrosine	0.12		0.12
Unknown (MST_1767)	0.14		0.12
Unknown (MST_1848.6)	0.10		
Unknown (MST_1967.6)	0.14		0.13
Unknown (RI_1130)	0.15	0.13	0.14
Unknown (RI_1145)	0.13		0.12
Unknown (RI_2240)	0.09		
Valine	0.15	0.12	0.15
Xylose		0.13	

Supplementary Table 4. Cell-pellet metabolites driving the separation in OPLS-DA models in alphabetic order (weighted loadings shown, $p<0.01$) comparing M1 and M3 for three types of cultures. Positive loadings correlated to M3 and negative loadings to M1

Metabolite	*Sa*	*Pa*	*Sa+Pa*
2-Methyl-2-Butanol		0.11	0.11
**4-Aminobutyric acid**	0.11		
6-Hydroxynicotinic acid	0.16		
Adenosyl-Methionine			0.11
**Arginine**	0.13		
B-Cyano Alanine	0.11	0.13	0.14
Beta-Alanine	0.15		
Cystathionine			0.12
Decanoic acid		0.10	
Ethanedioic acid der		0.11	0.13
Fumaric acid	0.12		
Galactose	0.12		
Glycerol	0.15	0.14	0.15
Homoserine	0.13	0.13	0.15
**Lysine**	0.13		
**Malic acid**		0.15	0.16
Myristic acid	0.15	0.14	0.16
Nonanoic acid			0.11
**Octadecanoic acid**		0.12	
Oxalic acid		0.12	0.13
**Phenylalanine**	0.14		0.11
**Ribose**			0.14
**Succinic acid**		0.10	0.14
Sucrose			0.12
Unknown (CONT_1442.5)		0.11	
Unknown (CONT_1684.3)		0.12	
Unknown (CONT_1827.2)		0.12	0.15
Unknown (CONT_1974.3)		0.14	0.13
Unknown (CONT_2187.3)		0.12	0.11
Unknown (CONT_2397)	0.13	0.14	0.15
Unknown (CONT-MST_1245)		0.12	
Unknown (Fluorescein, 5(6)-carboxy)	0.13		
Unknown (METB_408610)	0.15	0.11	0.14
Unknown (MST_1248.8)			0.13
Unknown (MST_1453.6)	0.14	0.14	0.16
Unknown (MST_1455.9)		0.14	0.13

Metabolite	*Sa*	*Pa*	*Sa+Pa*
4-Aminobutyric acid		-0.11	
Cadaverine		-0.11	
Fructose		-0.15	-0.17
Gluconic acid		-0.14	-0.13
Gluconic acid der	-0.15		-0.13
**Glucose**	-0.16		
Glutamic acid		-0.13	-0.13
Glycerolphosphate	-0.13	-0.11	
L-Aspartic acid		-0.12	
Lysine		-0.11	
Pyroglutamic acid		-0.15	-0.14
Ribitol	-0.17		
**Trehalose**	-0.11		
Unknown (CONT-MST_1245)	-0.12		
Unknown (MST_1659.6)		-0.13	
Unknown (MST_1770.9)	-0.11	-0.10	-0.10
Unknown (MST_1929.1)	-0.17		-0.13
Unknown (MST_1991)	-0.17	-0.12	-0.12
Unknown (RI_1282)	-0.12	-0.16	-0.16
Unknown (RI_1386)		-0.10	-0.13
Unknown (RI_1616)		-0.14	-0.12
Unknown (RI_1622)		-0.10	-0.11
Unknown (RI_1909)	-0.12		
Unknown (RI_1980)	-0.12	-0.16	-0.14
Uridine der	-0.17	-0.10	-0.13

Unknown (MST_1475.9)		0.12	0.11
Unknown (MST_1749.6)			0.14
Unknown (RI_1320)			0.12
Unknown (RI_1450)		0.14	0.15
Unknown (RI_1526)		0.13	
Unknown (RI_1876)	0.12	0.11	
Unknown (RI_1883)		0.15	0.16
Unknown (RI_1909)		0.12	
Unknown (RI_1909)			0.10
Unknown (RI_2011)		0.12	0.12
Xylibiose	0.16	0.10	0.13
